# Signal flow control of complex signaling networks

**DOI:** 10.1038/s41598-019-50790-0

**Published:** 2019-10-03

**Authors:** Daewon Lee, Kwang-Hyun Cho

**Affiliations:** Department of Bio and Brain Engineering, Korea Advanced Institute of Science and Technology (KAIST), 291 Daehak-ro, Yuseong-gu, Daejeon, 34141 Republic of Korea

**Keywords:** Cellular signalling networks, Network topology, Numerical simulations, Systems analysis

## Abstract

Complex disease such as cancer is often caused by genetic mutations that eventually alter the signal flow in the intra-cellular signaling network and result in different cell fate. Therefore, it is crucial to identify control targets that can most effectively block such unwanted signal flow. For this purpose, systems biological analysis provides a useful framework, but mathematical modeling of complicated signaling networks requires massive time-series measurements of signaling protein activity levels for accurate estimation of kinetic parameter values or regulatory logics. Here, we present a novel method, called *SFC* (*Signal Flow Control*), for identifying control targets without the information of kinetic parameter values or regulatory logics. Our method requires only the structural information of a signaling network and is based on the topological estimation of signal flow through the network. SFC will be particularly useful for a large-scale signaling network to which parameter estimation or inference of regulatory logics is no longer applicable in practice. The identified control targets have significant implication in drug development as they can be putative drug targets.

## Introduction

Transduction of abnormal signaling caused by genomic alterations induces cells to acquire the hallmarks of cancer^1–3^. Hence, controlling abnormal signaling is an important challenge in anti-cancer therapeutics^4^. However, the complexity of signaling networks makes us difficult to understand their dynamical behavior and discover effective control targets for overcoming drug resistance and preventing cancer relapse^5,6^. For instance, the unwanted elevation of AKT activity caused by inhibition of MEK is not intuitively understandable due to the complexity of the interconnected feedback loops and crosstalks in the ERK and PI3K signaling network^7^. In addition, even a simple signaling cascade composed of several biomolecules can be complex enough to be controlled, if a feedback regulation is involved in the cascade^8–11^.

To understand and analyze the behavior of complex signaling networks, we can adopt mathematical modeling^12,13^. Differential equation (DE) modeling provides us with a useful framework to reproduce real experimental observations and to investigate the underlying biological mechanism in a quantitative way^7,14–20^. However, DE modeling requires a lot of experimental data to accurately estimate kinetic parameter values in addition to the detailed information on nonlinear interaction relationships among biomolecules. These constraints usually confine DE modeling to relatively small-scale signaling networks. On the other hand, discrete-state dynamics (DD) modeling such as Boolean logic modeling has been demonstrated useful for qualitatively capturing essential dynamical properties of various biological systems from small to large-scale biological networks without such detailed information on kinetic parameter values^21–27^. However, DD modeling still requires the regulatory logics between biomolecules that should be collected by laborious and intensive curation from vast literature and databases or inferred from massive experimental measurements.

Control theories for complex networks including biological networks have been actively investigated recently^28–34^. Liu, Slotine and Barabási showed that we can control a variety of complex networks by controlling the driver nodes that are identifiable from the network structures^35^. Barabási and his colleagues also experimentally validated such structural controllability using the connectome of *Caenorhabditis elegans*. Fiedler and Mochizuki introduced a structure-based control theory using a feedback vertex set with which we can find out control targets that steer the nonlinear system to arrive at any desired attractor^36,37^. Zañudo and Albert devised a stable motif concept for Boolean network models, in which the node states in the stable motifs are fixed to achieve the desired steady state behavior^26,38^.

In this study, we developed a novel and practical control method, called *SFC* (*Signal Flow Control*), to find out control targets in a given signaling network, using only the network topology. Control methods based on DE or DD modeling require detailed information on kinetic parameter values or regulatory logics, which is a critical drawback in applying those methods to large-scale signaling networks^26,39^. The previous structure-based control methods for complex networks suggest too restricted solutions to achieve a specific control goal since they were developed for complete controllability^35,36,40^. To achieve a specific control goal using only the topological information of signaling networks, we devised a concept of ‘*influence*’ that describes how much the activity change of a particular signaling molecule affects the activity level of designated outputs that we want to control eventually. We applied the signal flow estimation algorithm^41^ that we developed in our previous study to predict the influence, and identified putative control targets that we can use for controlling the outputs of signaling networks. Interestingly, we found that the nodes distant from the output with higher influences can be more effective control targets in controlling the outputs than the proximal ones. The proposed SFC method can be used to reduce the search space for drug target discovery using only the topological information of large-scale signaling networks, and it would be particularly useful in practice when the kinetic parameter values or regulatory logics are not known.

## Results

### Definition of influence

The activity change of biomolecules with respect to time can be described by the following formula from the signal flow estimation algorithm^41^:

1 $$x(t+1)=\alpha Wx(t)+\beta b,$$

where *x*(*t*) ∈ R^*N*^ represents a set of activity levels of biomolecules at time *t* and *b* ∈ R^*N*^ represents the basal activity levels or signaling sources of biomolecules, respectively. *W* ∈ R^*N*×*N*^ is a link weight matrix where *W*_*ij*_ ∈ R is the weight of a link from a source node *j* to a target node *i*. The link weight represents how much the node *j* affects the node *i* through the link. Here, *W*_*ij*_ × *x*_*j*_ represents ‘signal flow’, showing how much the activity level of the source node *x*_*j*_ affects the activity level of its target node *x*_*i*_. *α*, *β* ∈ R are hyperparameters that adjust the proportion of signal flow and basal activity, respectively, in determining the activity level in the next step, *x*(*t* + 1). Perturbation such as inhibition or activation of a biomolecule by drug treatment or genetic mutations can be represented by changing *b* in Eq. (1).

To understand how the activity change of a source node affects the outputs through signal flow, we define ‘influence’. The influence, *S*_*ij*_, is basically the partial derivative of the output node *i* with respect to the activity change of a source node *j* as follows:

2 $${S}_{ij}=\frac{d{x}_{i}}{d{b}_{j}}=\frac{\partial {x}_{j}}{\partial {b}_{j}}\frac{d{x}_{i}}{d{x}_{j}},$$

where *x*_*j*_ and *x*_*i*_ represent the activities of the source node and the output node, respectively. *b*_*j*_ represents the basal activity or signaling source, to which the perturbation of *x*_*j*_ is reflected. In Eq. (2), influence is defined by the multiplication of local response (i.e., ∂*x*_*j*_/∂*b*_*j*_) and global response (i.e., *dx*_*i*_/*dx*_*j*_) of signaling molecule *i* when signaling molecule *j* is perturbed. *b*_*j*_ represents a perturbation that directly affects the activity of signaling molecule *j* (i.e., *x*_*j*_). Thus, the change in *x*_*j*_ caused by the perturbation in *b*_*j*_ is defined by the partial derivative, which means the only affected target by perturbation in *b*_*j*_ is *x*_*j*_ (i.e., local response of *x*_*j*_ with respect to *b*_*j*_). The change in the activity of signaling molecule *j* can affect another signaling molecule *i* through the intermediate signaling molecules in the network. Hence, *x*_*i*_ changed by *x*_*j*_ is defined by the total derivative, which means that we consider the global dynamics of all signaling molecules in the network to predict the change in *x*_*i*_ caused by *x*_*j*_ (i.e., global response of *x*_*i*_ with respect to *x*_*j*_). The influence is similarly defined as the global response to a perturbation in modular response analysis of signaling networks^42^ and the global correlation introduced for investigating the universality of network dynamics^43^. So, we can interpret ‘influence’ within the previous mathematical framework in view of the dynamics of complex networks to perturbations. However, our approach differs from any other previous approaches considering its application to the signal flow estimation that utilizes only the topological information of signaling networks.

**Figure 1:**
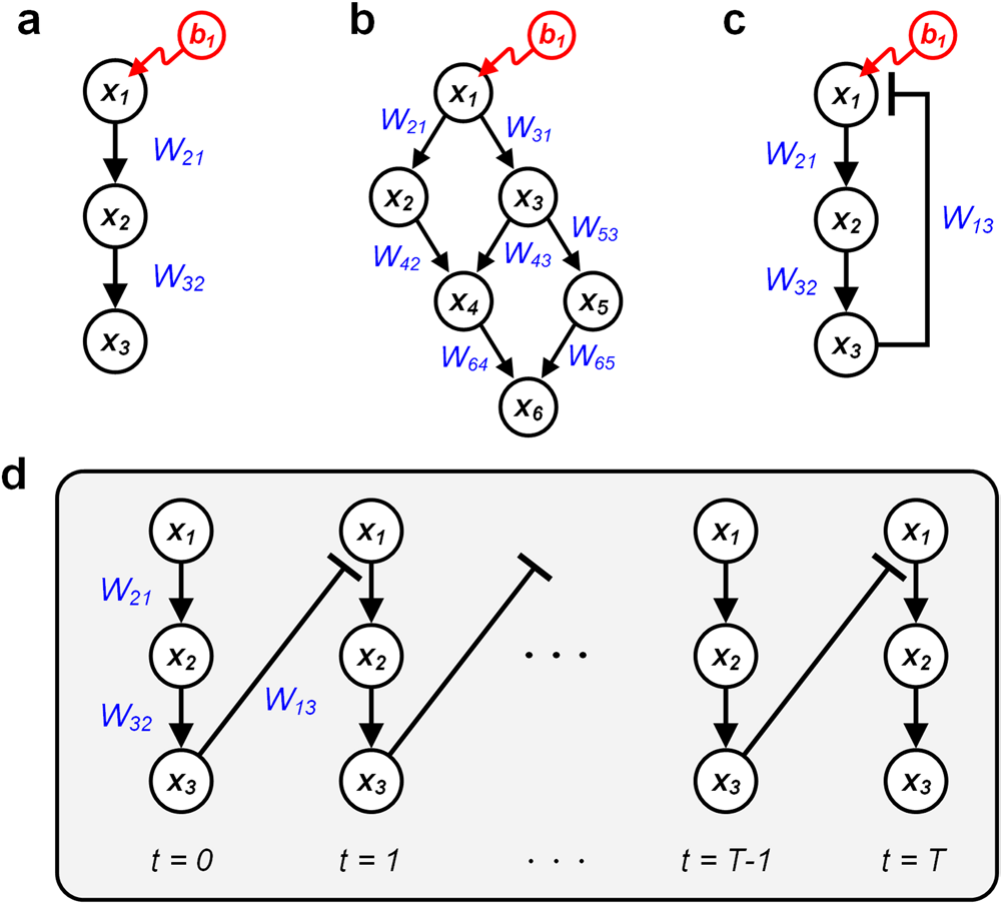
Examples illustrating the computation of the influence of a source node on the output. **(a)** 3-node cascade, **(b)** Multiple cascades with crosstalks, **(c)** 3-node negative feedback loop, **(d)** Unrolling of the 3-node negative feedback loop in (**c**).

Equation (2) can be computed for all pairs between source nodes and outputs, and the resulting matrix becomes an influence matrix, *S*. In a simple 3-node cascade (Fig. 1a), for instance, the influence of a source, *x*_1_ on node *x*_2_ at steady-state can be exactly described as a partial derivative of *x*_2_ with respect to *x*_1_ based on Eq. (1):

$$\begin{array}{rcl}{S}_{21} & = & \frac{d{x}_{2}}{d{b}_{1}}=\frac{\partial {x}_{1}}{\partial {b}_{1}}\frac{\partial {x}_{2}}{\partial {x}_{1}}\\  & = & \frac{\partial }{\partial {b}_{1}}(\beta {b}_{1})\frac{\partial }{\partial {x}_{1}}(\alpha {W}_{21}{x}_{1}+\beta {b}_{2})\\  & = & \beta \alpha {W}_{21},\end{array}$$

where *α, β* and *W* are those of Eq. (1). Note that the influence of node *x*_1_ on *x*_2_ depends on the weight of the link and the hyperparameters, *α* and *β*. On the other hand, the influence of node *x*_1_ whose signaling information reaches *x*_3_ through *x*_2_ is described as follows:

$$\begin{array}{rcl}{S}_{31} & = & \frac{d{x}_{3}}{d{b}_{1}}=\frac{\partial {x}_{1}}{\partial {b}_{1}}\frac{d{x}_{3}}{d{x}_{1}}=\frac{\partial {x}_{1}}{\partial {b}_{1}}\frac{\partial {x}_{2}}{\partial {x}_{1}}\frac{\partial {x}_{3}}{\partial {x}_{2}}\\  & = & \beta {\alpha }^{2}{W}_{21}{W}_{32}\end{array}$$

*S*_31_ is the multiplication of the two partial derivatives of *x*_3_ and *x*_2_ according to the chain rule of partial differentiation, resulting in the multiplication of two link weights on the path from *x*_1_ to *x*_3_.

The influence matrix, *S*, is essentially the summation of the multiplications of weights on all paths from the source node to the output node. In the example of multiple cascades with crosstalks (Fig. 1b), the influence of *x*_1_ on *x*_6_ is described as follows:

3 $$\begin{array}{rcl}{S}_{61} & = & \frac{d{x}_{6}}{d{b}_{1}}=\frac{\partial {x}_{1}}{\partial {b}_{1}}(\frac{\partial {x}_{2}}{\partial {x}_{1}}\frac{d{x}_{6}}{d{x}_{2}}+\frac{\partial {x}_{3}}{\partial {x}_{1}}\frac{d{x}_{6}}{d{x}_{3}})\\  & = & \frac{\partial {x}_{1}}{\partial {b}_{1}}(\frac{\partial {x}_{2}}{\partial {x}_{1}}\frac{\partial {x}_{4}}{\partial {x}_{2}}\frac{\partial {x}_{6}}{\partial {x}_{4}}+\frac{\partial {x}_{3}}{\partial {x}_{1}}\frac{\partial {x}_{4}}{\partial {x}_{3}}\frac{\partial {x}_{6}}{\partial {x}_{4}}+\frac{\partial {x}_{3}}{\partial {x}_{1}}\frac{\partial {x}_{5}}{\partial {x}_{3}}\frac{\partial {x}_{6}}{\partial {x}_{5}})\\  & = & \beta {\alpha }^{3}({W}_{21}{W}_{42}{W}_{64}+{W}_{31}{W}_{43}{W}_{64}+{W}_{31}{W}_{53}{W}_{65})\end{array}$$

Note that the multiplication of the three link weights in Eq. (3) corresponds to each path of length 3 in the network topology.

Computing the influence in a network with cyclic paths is more complicated than those of acyclic networks. In a simple negative feedback loop (Fig. 1c), expanding the paths from source node *x*_1_ to compute the influence has no limit. In this case, unrolling the loop helps us understand the computation procedure (Fig. 1d). As the longest path length approaches infinite, the influence at steady-state converges or diverges depending on the link weights and *α* following the Eq. (4).

4 $$\begin{array}{rcl}{S}_{31}^{T} & = & \mathop{\sum }\limits_{t=0}^{T}\frac{d{x}_{3}^{t}}{d{b}_{1}}=\mathop{\sum }\limits_{t=0}^{T}\frac{\partial {x}_{1}^{0}}{\partial {b}_{1}}\frac{d{x}_{3}^{t}}{d{x}_{1}^{0}}\\  & = & \beta \cdot (\frac{\partial {x}_{2}^{0}}{\partial {x}_{1}^{0}}\cdot \frac{\partial {x}_{3}^{0}}{\partial {x}_{2}^{0}}+\frac{\partial {x}_{2}^{0}}{\partial {x}_{1}^{0}}\cdot \frac{\partial {x}_{3}^{0}}{\partial {x}_{2}^{0}}\cdot \frac{\partial {x}_{1}^{1}}{\partial {x}_{3}^{0}}\frac{\partial {x}_{2}^{1}}{\partial {x}_{1}^{1}}\cdot \frac{\partial {x}_{3}^{1}}{\partial {x}_{2}^{1}}+\cdot \cdot \cdot +\frac{\partial {x}_{1}^{t=T}}{\partial {x}_{3}^{t=T-1}}\cdot \frac{\partial {x}_{2}^{t=T}}{\partial {x}_{1}^{t=T}}\cdot \frac{\partial {x}_{3}^{t=T}}{\partial {x}_{2}^{t=T}})\\  & = & \mathop{\sum }\limits_{t=0}^{T}\beta ({\alpha }^{2}{W}_{21}{W}_{32}){({\alpha }^{3}{W}_{13}{W}_{21}{W}_{32})}^{t}.\end{array}$$

In Eq. (4), we need to determine the unrolling parameter *T*. For instance, we can determine the value of *T* depending on the topological characteristics such as a multiple of the longest simple path length. The value of *T* should be appropriately determined to ensure that all signals properly propagate through the network.

The generalized formula of computing the influence matrix, *S*, considering both acyclic and cyclic networks is as follows:

5 $$S=\beta (I+\alpha W+{\alpha }^{2}{W}^{2}+\cdots +{\alpha }^{{L}_{M}}{W}^{{L}_{M}}),$$

where *L*_*M*_ is the longest path length. Eq. (5) can also be derived from the exact solution of Eq. (1).

6 $$\begin{array}{rcl}\frac{dx(t)}{db} & = & \frac{d}{db}\{\beta (I+\alpha W+{\alpha }^{2}{W}^{2}+\cdot \cdot \cdot +{\alpha }^{t-2}{W}^{t-2})b\}\\  & = & \beta (I+\alpha W+{\alpha }^{2}{W}^{2}+\cdot \cdot \cdot +{\alpha }^{t-2}{W}^{t-2}),\end{array}$$

where *x*(0) = 0 and *x*(1) = *βb*. Equations (5), (6) are essentially the same if we assume that both the longest path length *L*_*M*_ and time *t* approach infinity. The length of a cyclic path can be considered infinite, if we repeatedly count the path length along the cyclic paths. The infinity of time implies the system is at steady state. Hence, the computation of the influence in a network with cycles at steady state can be described as follows if Eq. (6) converges:

7 $${S}^{\ast }=\beta {(I-\alpha W)}^{-1}$$

If it is not possible to obtain S^*^ exactly, we can also utilize the iterative method with a limited number of iterations.

### Influence analysis of B2009 network

**Figure 2:**
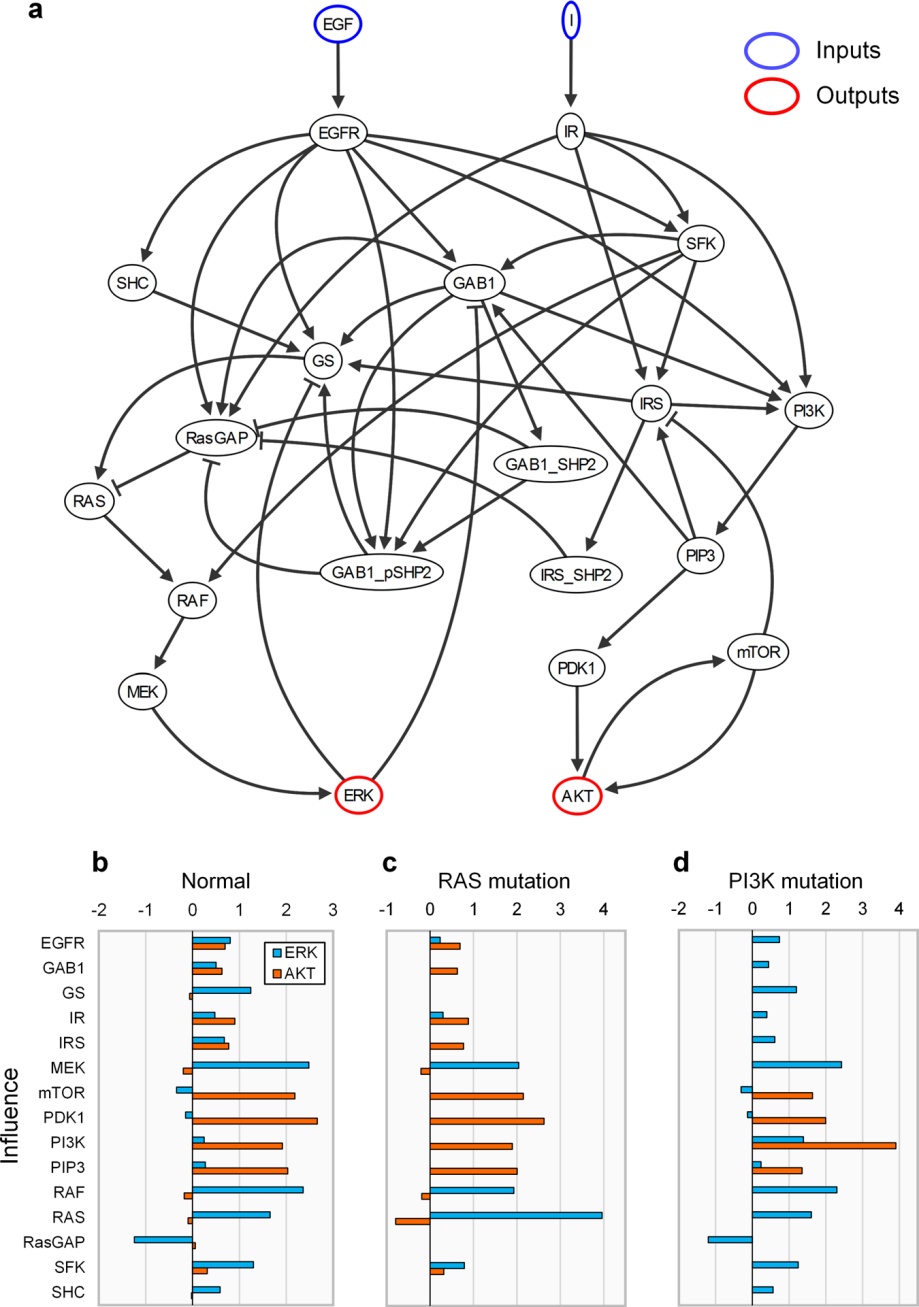
Analysis of influence in B2009. **(a)** Network topology of B2009, where EGF and insulin (I) are inputs (blue), and ERK and AKT are outputs (red). **(b)** Influence of each source node on ERK and AKT under the normal condition. **(c)** Influence under the condition of RAS mutation. **(d)** Influence under the condition of PI3K mutation. The bars represent the normalized values of influence (see **Methods** for details).

We analyzed the influence of source nodes on ERK and AKT in B2009 network (Fig. 2 and Table 1). Positive influence of a source denotes the activity of the output changes in the same direction with that of the source. Negative influence of a source denotes the activity of the output changes in the opposite direction with that of the source. Thus, it is required to find out the sources with positive influences if the control goal is defined to decrease the output activity using only the inhibitors of biomolecules. All nodes with positive influences on ERK, for example, are EGFR, GAB1, GS, IR, IRS, MEK, PI3K, PIP3, RAF, RAS, SFK, and SHC (Fig. 2b). The influence analysis suggests inhibiting these molecules could result in the decreased activity of ERK. However, inhibiting RasGAP is expected to increase the activity of ERK since the influence of RasGAP on ERK is negative. In the case of AKT, the positively influential node set includes IRS, PI3K, PIP3, PDK1, and mTOR, and the negatively influential node set includes GS, MEK, RAF, RAS, and SHC.

**Table 1:** Signaling networks used for validation of the algorithm.

Size	N	L	O	D	CPL	Description
Network						
B2009^15,41^	22	46	2	11	4.090	Signaling network of EGF-EGFR and insulin-IR, where the outputs are ERK and AKT. Control goal is to decrease the activities of both ERK and AKT. The inhibitory regulation of AKT against RAF was negligible in the original ODE model since the inhibition of RAF by AKT was found to be very weak in HEK293 cells^15^. We also found that the removal of the link between AKT and RAF improves the prediction accuracy of signal flow estimation algorithm in our previous study^41^. Hence, the link between AKT and RAF was removed from the original network.
S2015^47,66^	69	134	1	13	5.333	Signaling network of TGFβ-driven EMT, where EMT is the only phenotypic output node. Control goal is to decrease the activity of EMT node.
Z2015^38^	60	142	2	13	4.642	Signaling network of T-LGL leukemia survival, where the two phenotypic nodes of proliferation and apoptosis are defined as the outputs. Control goal is to decrease proliferation and increase apoptosis.
F2013^48^	97	253	2	12	4.641	Signaling network of colorectal tumorigenesis, where the two phenotypic nodes of proliferation and apoptosis are the outputs. We added ‘Proliferation’ node to the original model. Control goal is to decrease proliferation and increase apoptosis.
C2016^67^	200	696	3	13	5.198	Signaling network of colorectal tumorigenesis, where the three phenotypic nodes of proliferation, apoptosis, and metastasis are the outputs. We added ‘Proliferation’, ‘Apoptosis’ and ‘Metastasis’ nodes to the original model. Control goal is to decrease proliferation and metastasis, and increase apoptosis. We set all the ambiguous signs of 51 links as plus in this network.
T2016^68^	4388	15305	—	15	6.522	Literature-curated human signaling network. We obtained a subnetwork by identifying the giant component of the directed and signed links from the original database. No output was defined since the network was used to examine the performance of the algorithm.

**N**: number of nodes; **L**: number of links; **O**: number of outputs; **D**: network diameter (length of the longest shortest path); **CPL**: characteristic path length.

RAS and PI3K genes (e.g., KRAS and PIK3CA) often have constitutively activating mutations in human cancers^44,45^. Those genes having constitutively activating mutations are not affected by the upstream signal flows and their downstream signal flows are always amplified to a certain extent. So, we reflected the constitutively activating mutations to RAS or PI3K by adjusting the in- and out-link weights of each mutant protein in B2009 (see **Methods** for details). The influence distributions have been changed by the effects of the mutations (Fig. 2c,d). Under the condition of RAS mutation, GS, RasGAP, and SHC totally lose their influences on both of the outputs. However, GAB1, IRS, PI3K, and PIP3 retain their influences on AKT. Under the condition of PI3K mutation, the only three downstream nodes of PI3K (i.e., PDK1, PIP3, and mTOR) have influences on AKT, implying PI3K mutation blocks most pathways of signaling toward AKT in the network.

### Validation of influence as a predictive measure

To validate the influence as a predictive measure, we compared the analysis results of influence with the exact analysis results of the original ordinary differential equation (ODE) model of B2009. The median accuracies for predicting the direction of the activity change for ERK and AKT using the signs of influence are between 0.8 and 0.9 over the 200 sub-datasets of B2009 (Supplementary Fig. S1a). We have also investigated which nodes are difficult to be predicted on their directions of activity change using the influence (Supplementary Fig. S1b). It turns out that the results of mTOR perturbation are the most difficult to be predicted. This is because some link weights related with the function of mTOR-IRS feedback are not appropriately estimated using only the network topology^41^.

We also calculated Pearson correlation coefficient between the influence and the log-activity change of the original ODE model to examine whether the influence can be a measure for prioritizing biomolecules (Supplementary Fig. S1c). The influence is positively correlated with the activity change of the original ODE model under the perturbation of each target, showing that we can utilize the influence as a measure to identify the most influential nodes.

### Discovery of control targets in B2009 network

**Figure 3:**
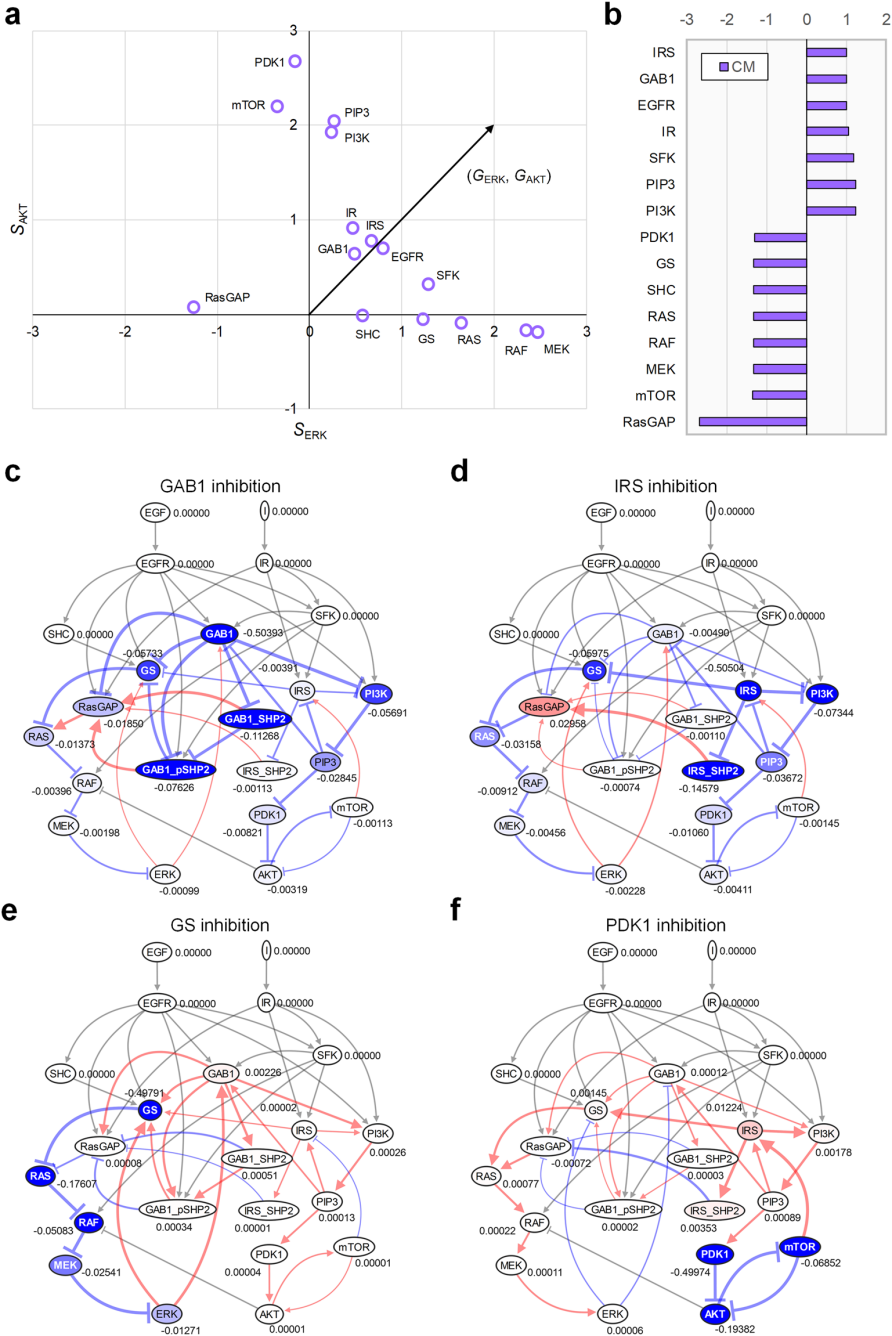
Controlling ERK and AKT based on the influence analysis. **(a)** 2D influence space of ERK and AKT in B2009. *S*_ERK_ and *S*_AKT_ represent the influence of each node on ERK and AKT, respectively, in Fig. 2. (*G*_ERK_, *G*_AKT_) denotes the direction of the control goal state. **(b)** Composite measure (CM) is defined and used to examine how close each influence state is to the desired state of control goal (see **Methods** section for the definition of CM). Smaller with positive sign is better. **(c–f)** The changes in activity levels and signal flows after the inhibition of **(c)** GAB1, **(d)** IRS, **(e)** GS, and **(f)** PDK1.

The control goal for B2009 network in this study is assumed to simultaneously decrease the activities of ERK and AKT through inhibiting a single target molecule. To measure how close each influence vector, (*S*_ERK_, *S*_AKT_), is to the goal state in the two dimensional space of the influence (Fig. 3a), we defined the composite measure (CM) in consideration of the cosine distance to the goal state and the direction of the influence vector (see **Methods** for details). Promising control targets should have the same sign of influences on the two outputs (i.e., positive influences on ERK and AKT) since inhibiting a target should reduce the activities of the two outputs simultaneously. For instance, the CM indicates GAB1 and IRS are promising control target candidates that can simultaneously decrease the activities of ERK and AKT, whereas GS and PDK1 appear to be undesirable targets that oppositely control the two outputs (Fig. 3b). To demonstrate the activity patterns of the outputs after controlling the targets, we performed the signal flow analysis^41^. Inhibition of GAB1 or IRS has been shown to successfully suppress both ERK and AKT (Fig. 3c,d), whereas inhibition of GS or PDK1 fails to suppress the two outputs simultaneously (Fig. 3e,f). Computing the composite measure under the condition of RAS mutation or PI3K mutation shows that some target candidates have no effect due to the constitutively activating mutation, which blocks signal flows toward the outputs (Supplementary Fig. S2). SFK and PIP3, for example, are promising control targets for RAS mutation and PI3K mutation, respectively. In the case of RAS mutation, SFK has the positive smallest CM (i.e., the smallest cosine distance to the goal state with the same signs of influences on the two outputs), so it is the only one promising control target if we exclude the receptors, EGFR and IR, from the target candidates. In the case of PI3K mutation, PIP3 is the only one promising control target as in the case of RAS, if we exclude the mutated PI3K from the target candidates. These results suggest that we can discover and prioritize target candidates to achieve control goals by utilizing the influence as a measure.

### Discovery of control targets in S2015 network

**Figure 4:**
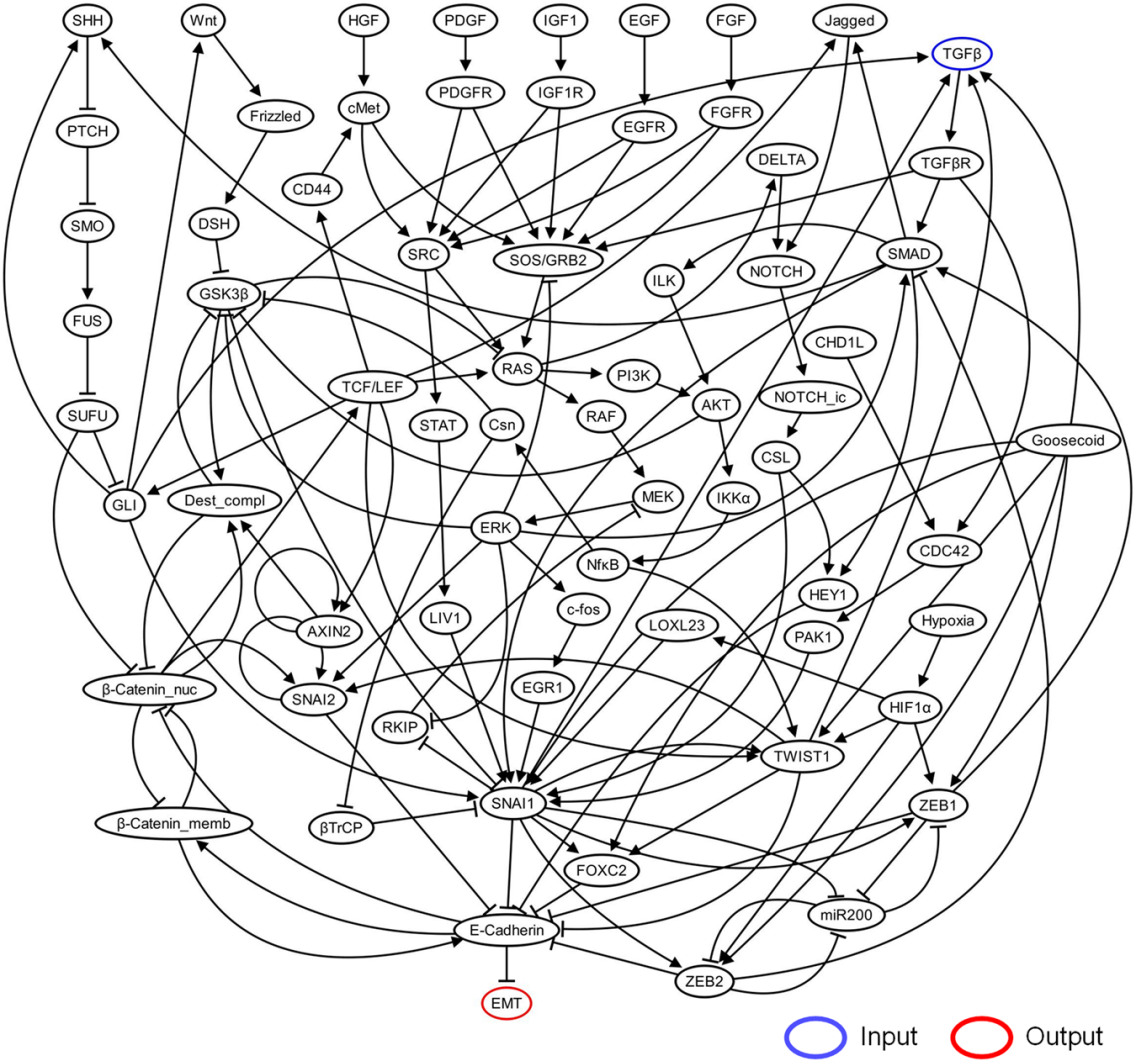
Network topology of TGFβ-driven EMT in S2015. TGFβ is input (blue), and EMT is output (red). The number of nodes and the number of links are 69 and 134, respectively.

**Figure 5:**
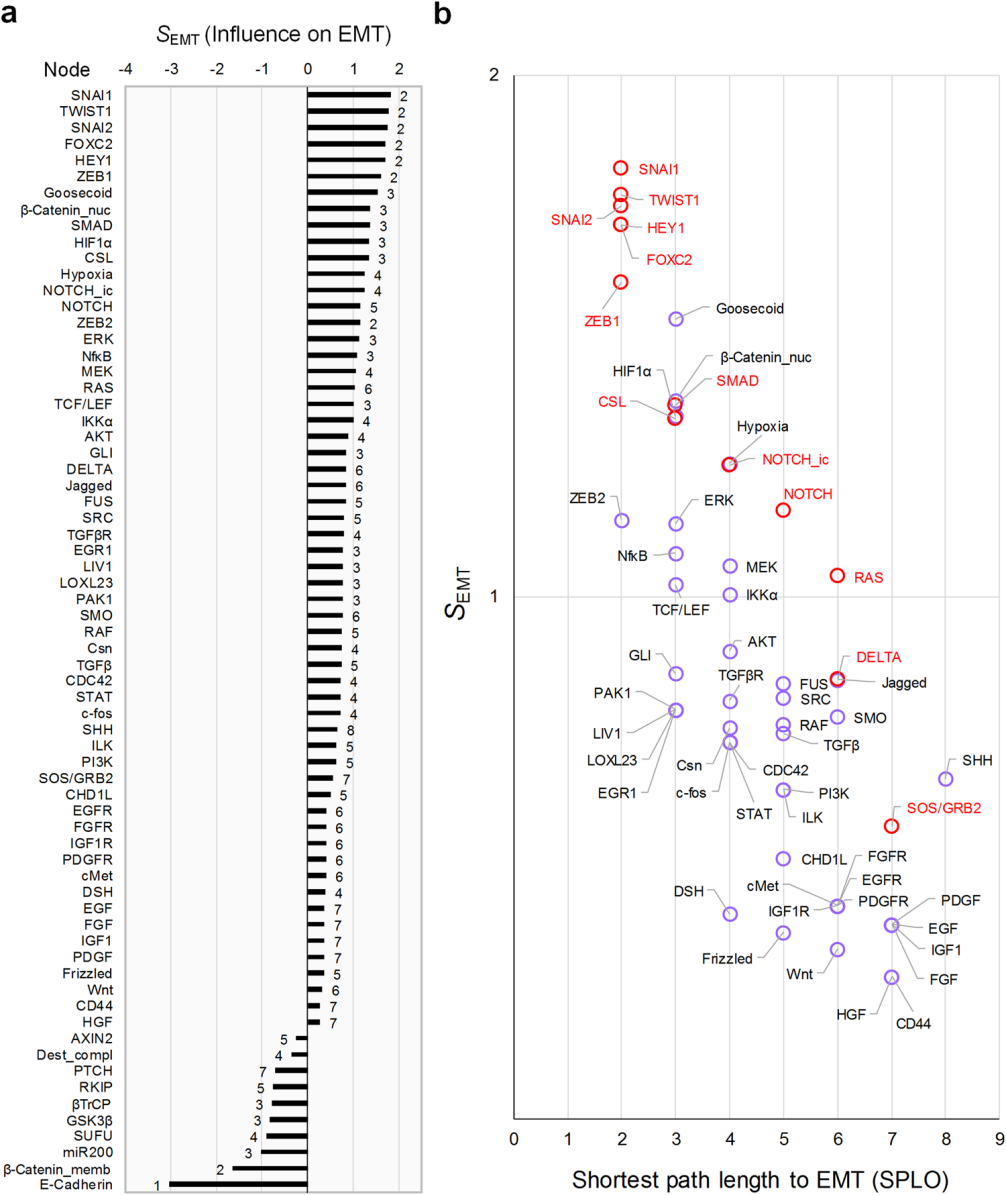
Analysis of influence in S2015. **(a)** Influence of each node on EMT. Each number beside the bar denotes the shortest path length of each node to the output, EMT (SPLO). **(b)** Scatter plot of the positive influence with respect to SPLO in (**a**). Red labels denote the control targets which are included in the single or dual control node set that can fully suppress EMT in the original Boolean model of S2015.

We further analyzed a relatively more complex network, the TGFβ-driven epithelial-mesenchymal transition (EMT) network of S2015 composed of 69 nodes and 134 links with multiple feedbacks and crosstalks (Fig. 4 and Table 1). The top-10 most influential nodes include SNAI1, SNAI2, TWIST1, FOXC2, HEY1, ZEB1, and β-Catenin_memb, which can regulate the only direct regulator of EMT phenotype node, E-Cadherin (Fig. 5a). The shortest path length to the output (SPLO) is negatively correlated with the absolute value of the influence (Pearson correlation coefficient is −0.753), implying that the proximal nodes tend to have higher influence. However, there are some exceptional nodes that do not follow this tendency. For instance, ZEB2 is the direct regulator of E-Cadherin with SPLO = 2, but the rank of its influence is 15, which is relatively lower than those of the other nodes with SPLO = 2. It is because ZEB2 negatively regulates SMAD, one of the highly influential nodes on EMT, through the cascade of SNAI1 and E-Cadherin, while it positively regulates EMT through E-Cadherin in the incoherent feedforward loop (I-FFL) (Fig. 4)^46^. Removing the link from ZEB2 to SMAD results in the rank of ZEB2 included in top 10 (Supplementary Fig. S3a), implying the positive influence of ZEB2 on EMT in the intact network decreases due to the ZEB2-SMAD inhibitory axis in the I-FFL. On the other hand, removing the link from ZEB2 to E-Cadherin dramatically decreases the influence of ZEB2, where even the sign of the influence is changed to negative (Supplementary Fig. S3b), implying the cascade of ZEB2 and E-Cadherin to EMT in the intact network plays a role as the activating axis in the I-FFL. These results suggest that the influence analysis can capture the role of complex network topology such as the feedforward loop structure including ZEB2, SMAD, and E-Cadherin (Supplementary Fig. S4).

We found that the effective control targets discovered in the original Boolean model of S2015 are highly influential even though they are not proximal to the output^47^. NOTCH and RAS, for instance, are relatively far from the output, but have higher influence than those with the same SPLO (Fig. 5a). Hence, we analyzed the relationship between SPLO and the influence of the effective control targets of the original model. Interestingly, the original control targets tend to have high influence amongst the group of targets having an identical SPLO (Fig. 5b, red labels). Based on this result, we identified Goosecoid, β-Catenin_nuc, HIF1α, Hypoxia, Jagged, and SHH as promising control targets, which were considered relatively less effective in the original model with respect to single or dual inhibition control. There are experimental evidences supporting that controlling each of these targets actually inhibits EMT (Table 2).

**Table 2:** Experimental evidences supporting the effectiveness as potential drug targets for the identified control targets.

Target node	Description	References
Goosecoid	The knockdown of Goosecoid changed the expression pattern of EMT markers in hepatocellular carcinoma (HCC) cell lines, where E-cadherin was increased and vimentin was decreased. In addition, patients with highly expressed Goosecoid in HCC showed poor survival rate and earlier lung metastasis.	^69^
β-Catenin_nuc	Nuclear β-catenin induces EMT through LEF-1 transcriptional activity in epithelial colorectal cancer (CRC) cells.	^70^
	Nuclear β-catenin regulates the expression of the mesenchymal genes in Twist2-induced EMT of ovarian cancer.	^71^
	The inhibition of β-catenin binding to Smad3 to form a transcriptional complex blocked TGF-β1-driven EMT in renal tubular epithelial cells, irrespective of β-catenin/LEF-1.	^72^
	IWR-1, a small molecule inhibitor, promotes the degradation of β-catenin, and thereby inhibits TNFα-driven EMT in CRC cell lines.	^73^
HIF1α & Hypoxia	HIF-1α promotes EMT through ZEB1 in CRC.	^74^
	Hypoxia stimulates TGF-β-driven EMT through HIF-1α in mouse hepatocytes. Mesenchymal markers such as vimentin or fibroblast-specific protein-1 (FSP-1) were upregulated in the mouse hepatocytes exposed to room air or 1% oxygen.	^75^
Jagged	Inhibiting the expression of Jagged-1 prevented TGF-β2-induced EMT in retinal pigment epithelium (RPE) cells.	^76^
SHH	The inhibition of the SHH signaling pathway by cyclopamine blocked SHH-driven EMT in renal tubular epithelial cells (RTEC). TGF-β1 stimulation in RTECs increased the expression of SMO and GLI1, while it decreased the expression of PTCH1.	^77^
	TGF-β1-induced EMT, that was associated with increased cell motility, invasiveness, and clonogenicity, was attenuated by inhibiting SHH in bladder cancer cells.	^78^

**Figure 6:**
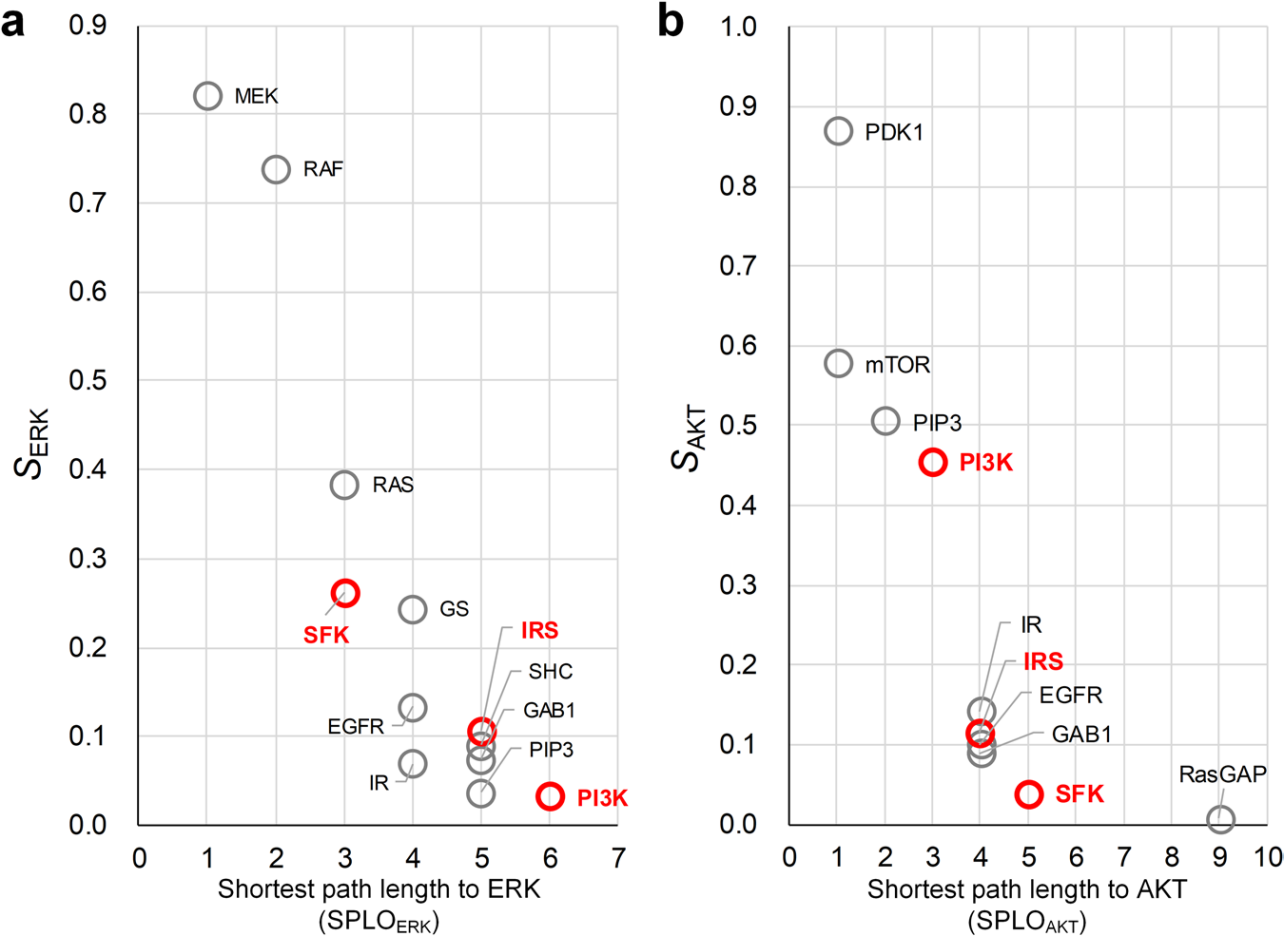
Influence versus SPLO in B2009. Scatter plot of the positive influence of each node on **(a)** ERK and **(b)** AKT with respect to SPLO. Red labels denote the common promising control targets between ERK and AKT, which were discovered by obtaining C_ERK_ ∩ C_AKT_, where C_*i*_ is the set of the top-2 most influential nodes on output node *i* in each group of SPLO.

We also analyzed the network of B2009, where the two most influential nodes in each group of SPLO were selected as promising control targets. The common target candidates for controlling the two outputs were IRS, SFK, and PI3K (Fig. 6), among which IRS was discovered as the most promising control target using the composite measure (Fig. 3). These results suggest that we can enhance the possibility of discovering more promising control targets by analyzing the influence as well as SPLO.

### Discovery of control targets using SI-plot

**Figure 7:**
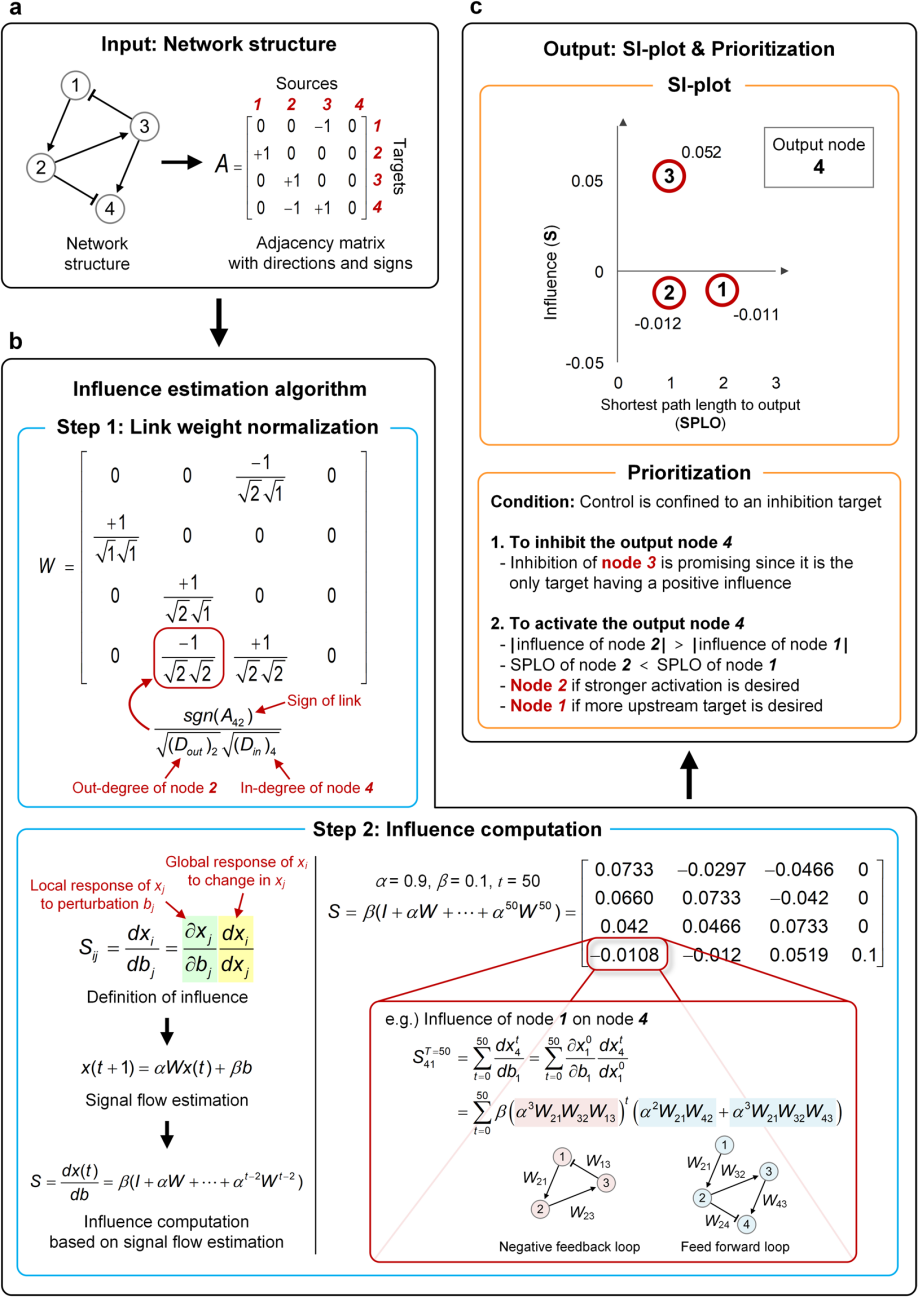
Workflow of SI-plot analysis. **(a)** The only input to the algorithm is the structure of a signaling network represented by an adjacency matrix with directions and signs. **(b)** The influence of node *j* on node *i* (*S*_*ij*_) is estimated using only the input adjacency matrix. The first step is the normalization of link weights, in which each link weight is determined by averaging the degrees of source and target nodes. The second step is the computation of influence based on the signal flow estimation algorithm. *S*_*ij*_ consists of the local response of node *j* to the perturbation of node *j* (*∂x*_*j*_/*∂b*_*j*_) and the global response of node *i* to the change in node *j* (*dx*_*i*_/*dx*_*j*_). **(c)** The output is an SI-plot, which is obtained by plotting the influence against SPLO. Promising control targets can be discovered by prioritizing the nodes in the SI-plot according to certain criteria such as a set of the top-*k* or top-*n*% most influential nodes on the output in each SPLO group.

Based on our observation in B2009 and S2015, we developed an analysis based on SPLO-influence-plot (SI-plot) (Fig. 7). The only input to SI-plot analysis is the topology of a signaling network, represented by an adjacency matrix with directions and signs (Fig. 7a). The directions denote the source and target nodes of links, and the signs represent the regulation types of links. For instance, positive and negative signs represent activation and inhibition regulations, respectively. The first step of the influence estimation is the normalization of link weights, in which each link weight is determined by averaging the degrees of source and target nodes (e.g., the geometric mean of the out-degree of the source and the in-degree of the target) (Fig. 7b, Step 1). The link weight normalization in SI-plot analysis is the same as that of signal flow estimation algorithm^41^. The second step is the computation of influence based on Eq. (5) (Fig. 7b, Step 2). Thereafter, we obtain an SI-plot that is a plot of the influence against SPLO (the process of computing SPLO is omitted for brevity in Fig. 7). To find out promising control target candidates, we prioritize the nodes in SI-plot according to specific criteria (Fig. 7c). In the toy example of Fig. 7, for instance, the only available control target is node 3, if the control goal is to decrease the activity of the node 4 by inhibiting the control target. However, there are two control target candidates, if the control goal is to activate node 4. In this case, we need to define specific criteria to select the control target. For instance, node 2 might be promising if we require stronger activation of the output, while node 1 might be promising if more upstream target is desired. In this study, we assumed that more influential nodes in each SPLO group are likely to be control target candidates in SI-plot.

To validate SI-plot in identifying control target candidates, we performed SI-plot analysis for the signaling networks of various sizes in addition to B2009 and S2015 (Table 1). Z2015 is T-LGL leukemia survival signaling network, in which two phenotypic nodes, proliferation and apoptosis, are defined as the outputs (Table 1). As a result, we found a subset of the original control targets using SI-plot, when the control goal in Z2015 was defined to decrease proliferation and increase apoptosis. Fas, Ceramide, TBET, ERK, MEK, and GRB2 are found as highly influential in promoting apoptosis in SI-plot (Supplementary Fig. S5, red in the top 3 of each group). On the other hand, sFas, S1P, PDGFR, and SPHK1 are expected to effectively suppress apoptosis in the SI-plot (Supplementary Fig. S5, red in the bottom 3 of each group). For proliferation, IL2RB, PI3K, RAS, and GRB2 are also highly influential in promoting proliferation as in the original model (Supplementary Fig. S6, red in the top 3 of each group). On the other hand, S1P and SPHK1 are found to suppress proliferation as in the original model (Supplementary Fig. S6, red in the bottom 3 of each group). Interestingly, these control target candidates are also included in the control targets suggested by the previous study on stable motif control and further supported by experimental evidences^38^. If the control goal in Z2015 is defined to increase apoptosis without enhanced proliferation by inhibiting a single target, the promising control target candidates are BclxL, MCL1, sFas, P2, PDGFR, and GPCR. Although A20, S1P, and SPHK1 are highly influential in suppressing apoptosis, they also promote proliferation, suggesting that inhibition of these targets can enhance proliferation and thereby result in a bypass pathway for drug resistance. On the other hand, if we want to decrease proliferation by increasing apoptosis through inhibiting a single target in Z2015, the promising control target candidates are IL2RB, NFKB, PI3K, TPL2, TAX, and PDGF. However, inhibiting one of RANTES, IL2RA, NFAT, TNF, RAS, PLCG1, GRB2, IL2, and IL15 can decrease apoptosis, implying that they can facilitate cell survival and thereby cause drug resistance in the leukemia. From this analysis, we suggest control target candidates that are different from those of original model. For instance, targeting one of PLCG1, NFAT, RANTES, and IL2RAT is expected to increase apoptosis without enhancing proliferation. Moreover, targeting one of TAX, TPL2, PDGF, and NFKB might decrease proliferation without reducing apoptosis in Z2015.

We also analyzed the signaling networks of colorectal tumorigenesis with different sizes using SI-plot (Table 1, F2013 and C2016), and discovered the subsets of the original control targets. The network of F2013 for colorectal tumorigenesis consists of 97 nodes and 253 links, in which the outputs are two phenotypic nodes, proliferation and apoptosis. We found 14 targets out of the 18 original targets (i.e., 78%): AKT, Caspase8, Caspase9, cdc20, CyclinB, CyclinD, CyclinE, E2F, FADD, p21, p27, PDK1, Rb, and Smad, when the control goal was to decrease proliferation and metastasis, and increase apoptosis (Supplementary Figs S7, S8). These control targets were originally suggested through a full search of all possible interventions based on nonlinear discrete dynamics model with detailed regulatory logics in F2013^48^. In addition, we found out Bcl-XL as a novel target for increasing apoptosis without enhancing proliferation, COX412 and GSH as novel targets for decreasing proliferation without reducing apoptosis. We also analyzed the colorectal cancer network of C2016, in which apoptosis, proliferation, and metastasis nodes are defined as the outputs among 200 nodes with 696 links. Cho *et al*. found 8 targets to drive any state of colon cancer cells toward quiescent or normal proliferative phenotypic states using a genetic algorithm for optimization. Intriguingly, we also discovered 6 targets out of the 8 original targets (i.e., 75%) using SI-plot analysis in C2016: β-catenin, ERK, MEK, PP2A, Smad2/4, and Snail (Supplementary Figs S9–S11). We also found novel control target candidates based on SI-plot for increasing apoptosis without enhancing metastasis and proliferation: A20, AC, Bcl2, DGK, GAB1, IκB, MKK3, MKK6, MKP, p14arf, p38, Rb, RhoGDI, TAO1/2, and XIAP, and for decreasing both metastasis and proliferation without reducing apoptosis: IKK, RAP1, RAS, and TAB1/2.

### Robustness of SI-plot analysis against incomplete network topology

One of the critical problems for utilizing the topology of a signaling network is that there is no complete information on the topology in many cases. Thus, we need to investigate how robust the SI-plot analysis is with respect to the uncertainty of the topological information of a given network. To consider uncertainties in network topologies, we defined a ‘structural perturbation’ by randomization of a given network topology through degree-preserving link swapping and sign flipping. To quantitatively measure the robustness with a statistical significance, we also performed an enrichment analysis based on hypergeometric test in which the sample is a set of the control target candidates found by SI-plot analysis and the population is all the nodes in a given network. We chose the network of C2016 for robustness analysis (see **Methods** for details).

**Figure 8:**
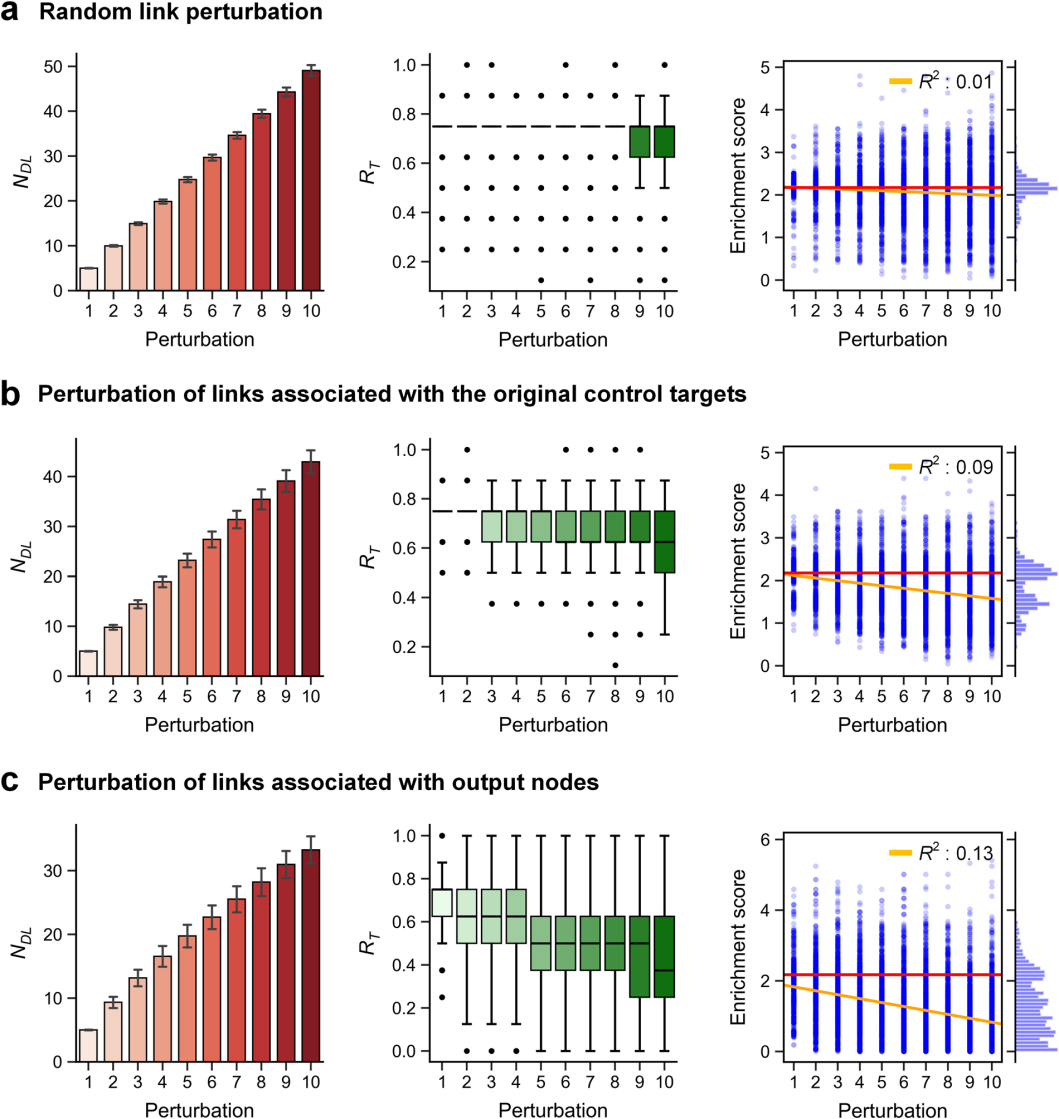
Robustness of SI-plot analysis against structural perturbation in C2016. **(a)** Random link perturbation. Links of the structural perturbation are randomly selected. **(b)** Perturbation of links associated with the original control targets. One of the links of the original control targets is randomly selected in each structural perturbation. **(c)** Perturbation of links associated with output nodes. One of the links of the output nodes is randomly selected in each structural perturbation. Structural perturbation denotes the randomization of the network topology through degree-preserving link swapping and sign flipping. *N*_*DL*_ is the number of new links different to the links of the original network. *R*_*T*_ is the ratio of the number of the original control targets that are found by SI-plot analysis to the total number of the original control targets. Enrichment score indicates the ability of SI-plot analysis to discover the original control targets with a statistical significance (see **Methods** for details).

To measure the robustness of SI-plot analysis for C2016, we investigated the ratio of the number of the original control targets that are found by SI-plot analysis to the total number of the original control targets (denoted as *R*_*T*_) while increasing the structural perturbation. We repeated each perturbation simulation 1,000 times (i.e., the total number of simulations is 10,000). *R*_*T*_ of no perturbation condition (0.75) was relatively well maintained even when the perturbed network contains about 40 different links to the original network (Fig. 8a, left and middle panels). The mode of enrichment score distribution was almost close to that of no perturbation condition (−*log*_10_(6.699E-3)) over the perturbations (Fig. 8a, right panel).We conjectured the arbitrary perturbation of links might have little effect on the influence and SPLO. So, we further investigated how robust the SI-plot analysis is if the links of critical nodes, such as the original control targets or the outputs, are always included in the perturbations. Intriguingly, perturbing the output nodes showed greater reduction of robustness than perturbing the original control targets (Fig. 8b,c). The median of *R*_*T*_ decreased more dramatically and the variance of *R*_*T*_ became much larger in the case of perturbing the outputs than perturbing the original control targets. These results imply that the robustness of SI-plot analysis might be more vulnerable to incorrect information on the regulatory relationships around the outputs of signaling networks, if they are critical in determining the cell fate.

### Performance optimization

To optimize and test the performance of the influence estimation algorithm, we also implemented the GPU version of the algorithm (see **Methods** for details). The time complexity of the naïve implementation of the algorithm is *O*(*n*^3^), which is characterized by the weight matrix multiplication in the main loop (Table 3). Hence, we expected that the optimization based on GPU would be promising to improve the performance of the algorithm. In T2016 network that is a large-scale signaling network (Table 1, Supplementary Fig. S12a), we obtained about 45x performance improvement against the CPU version (Supplementary Fig. S12b). To test the scalability of the GPU optimization, we generated Erdős–Rényi (binomial degree distribution) and Barabási–Albert (power-law degree distribution) random networks with different sizes. Intriguingly, the GPU optimization resulted in more about 40–70x performance improvement for the large-scale random networks (Supplementary Fig. S13). These results imply that the optimized algorithm can be used in the future for analyzing genome-scale networks that are established from a cohort of cancer patients. For instance, it might require only 3 days for processing 10,000 patients using the 8 GPU devices, whereas 107 days might be required by the 8 CPUs.

**Table 3:**
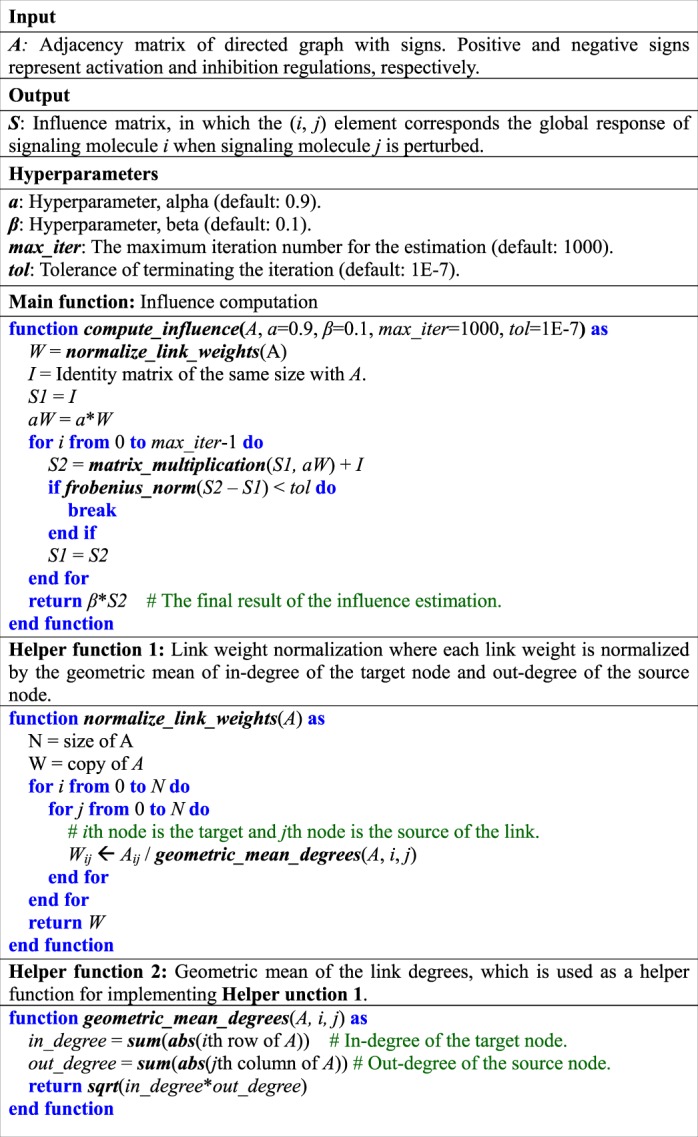
Pseudo-code for analyzing the influence in SFC.

## Discussion

Recently, a few studies showed the possibility of predicting the perturbation effects in signaling networks based only on the topological information of networks^49–51^ including our previous study^41^. Such possibility of estimating signal flows motivated us to develop the presented method of controlling signal flows using only the network topology. In this study, we have developed a very practical method, called SFC, to control the signal flow of complex signaling networks. By introducing the concept of influence to the network topology, we were able to identify control target candidates such that the activity levels of the output nodes to be controlled in a desired direction to achieve specific control objectives.

**Figure 9:**
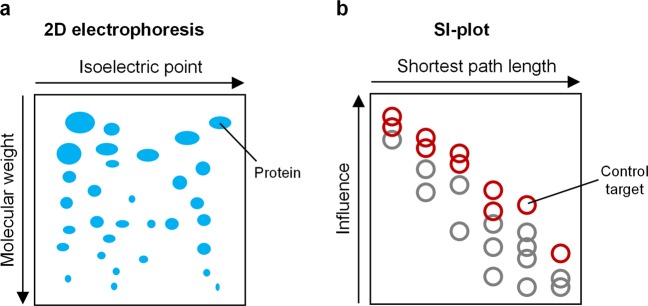
Conceptual diagrams illustrating the two-dimensional information of SI-plot. **(a)** 2D electrophoresis enables us to distinguish and identify specific proteins of the same molecular weight by utilizing the two-dimensional information given by molecular weight and isoelectric point. **(b)** Similarly, in SFC based on SI-plot, we can discover control target candidates more efficiently by analyzing the two-dimensional information represented by the influence and the shortest path length. This is a conceptual advancement comparable to the invention of 2D electrophoresis.

Intriguingly, analysis of the influence with SPLO in SI-plot of SFC enabled us to discover the control target candidates that are difficult to be found by investigating only the influence or the shortest path length alone. It is often considered that nodes nearby the output (nodes with small SPLO) can be effective control targets. For instance, RAF or MEK can be considered as effective control targets to downregulate ERK since they are the nearest upstream regulators of ERK in the signaling network^52^. However, we showed that distant nodes with high influences can be more effective control targets than the proximal nodes in controlling the outputs in a desired direction. This counterintuitive result was well exemplified by the cases of ZEB2 and RAS in the EMT regulatory network (Fig. 5), IRS in the signaling network of EGF-EGFR and insulin-IR (Fig. 6), MEK and GRB2 in the signaling network of T-LGL leukemia survival (Supplementary Figs S5, S6), p21 and PDK1 in F2013 (Supplementary Figs S7, S8), and MEK in C2016 (Supplementary Figs S9–S11) colorectal cancer networks. We believe that the conceptual advancement of SI-plot is comparable to that of two-dimensional electrophoresis^53^ (Fig. 9). In both methods to identify specific objects, integrating the two dimensional information allows us to achieve greater results than using only one dimensional information.

There are limitations in our approach. For instance, the synergistic effects of drug target combinations are difficult to be predicted based on the estimated influence in SFC since the synergistic effects are usually caused by the nonlinear dynamical interaction of biomolecules^54–56^. One way of overcoming this limitation is identifying a small set of promising candidates using the SI-plot of SFC and then experimentally searching for the synergistic effects among the possible combinations of targets within the set. Another limitation lies in the incomplete curation of network topology and the resulting inaccurate estimation of signal flows^41^. We found that an inaccurate information on the links associated with the output nodes can deteriorate the robustness of SI-plot analysis (Fig. 8c). To overcome this limitation, we can combine various computational methods of inferring and curating biological networks and thereby complement inaccurate information on network topology^17,41,57,58^. Although we have implemented mutation effects in B2009, F2013 and C2016, it is difficult for our approach to capture a subtle difference between the effects of different mutations on the same signaling molecule since our approach utilizes only the topological information of signaling networks. Reflecting such effects of different mutations on the same signaling molecule would be one of future challenges to improve our signal flow control algorithm.

We could discover control target candidates by collecting a set of the top-*k* or top-*n*% nodes from each SPLO group in SI-plot analysis. However, there is no theoretical criteria for determining the parameters, *k* or *n*, for a given signaling network, and therefore we need to set those values empirically. To complement this empirical aspect of SI-plot analysis and generalize the criteria to discover the control target candidates, we can employ statistical criteria such as hypergeometric test as in the robustness analysis. For instance, if the statistical significance of the results in SI-plot analysis is too low, we can adjust the parameters, *k* or *n*, until we meet a certain statistical significance. In C2016, for instance, the p-value of hypergeometric test is 6.699E-03, and we can reject the results with p-values greater than 6.7E-03 to find out a novel set of control target candidates in C2016.

If a signaling network has no phenotypic output node at all, outputs and control goal need to be explicitly defined such that SI-plot analysis can be applied to the network. For instance, if researchers want to find out some highly influential target proteins to control the activity of a specific protein *A*, we can define the output as protein *A* in the network. However, if a signaling network is too large and complex to properly define outputs, we can utilize other methods that suggest some important markers related to a specific biological phenomenon or disease. For instance, we can select hallmark gene sets of metastasis^59^ and define them as the outputs of a large-scale cancer network to discover the control targets that can control metastasis.

We expect that SFC can be widely used as a very practical method for discovering drug targets when parameter estimation or inference of regulatory logics is uneasy to be done. We note that other methods based on mathematical modeling such as DE or DD require extensive time-series measurements and time-consuming simulations for optimization or *a priori* information on kinetic parameters and interaction logics even if they might be useful in capturing the detailed nonlinear dynamical characteristics of signaling networks. On the other hand, machine learning approaches such as deep learning demand massive data for training and, more importantly, lack an explanation about the underlying biological mechanism even though they can achieve high performance in predicting drug responses since they are basically a kind of black-box modeling. In contrast, SFC can be a practical compromise that complements the lack of kinetic parameter information using the network topology, performs in a feasible time with polynomial complexity, explains the underlying mechanism by signal flow analysis, and provides promising outcomes that can reduce any unnecessary wet experiments. Consequently, SFC is expected to be a useful method that can initially reduce the search space of drug target candidates by filtering out all non-influential nodes if the topological information of a signaling network is available.

## Methods

### Signaling networks

We have chosen the signaling networks described by ODE or discrete logic models (Table 1) for which the original control targets discovered and validated. Based on the original control targets, we can evaluate our approach by investigating whether the original control targets can also be found by our signal flow control (SFC) algorithm using only the topological information of the signaling networks. SFC algorithm can be applied to any kind of signaling networks with directions (i.e., source and target) and signs (i.e., activation or inhibition) where output nodes are defined for computing the influence and SPLO.

### Influence analysis

We can analyze the influence based on Eq. (5) using an iterative method. For the detailed procedure of computing the influence, we provide a pseudo-code of the computation algorithm in Table 3. The condition for terminating the iteration can be set, for instance, as ||*S*(*t* + 1) − *S*(*t*)||_F_  < 1E-6, where ||∙||_F_ denotes Frobenius norm. We set the hyperparameter values as *α* = 0.9 and *β* = 0.1, unless specified otherwise. We also applied the normalization of link weight matrix as in the signal flow estimation of the previous study^41^.

### Validation of the estimated influence as a predictive measure

We used 200 sub-datasets of the original ODE model of B2009 to validate whether the influence can be used as a predictive measure. The sub-datasets were generated by performing extensive simulations under various conditions with respect to input stimulation, simulation time, and the type of activity measurement (i.e., the area under curve) in our previous study^41^.

### Analysis of influence in B2009

To discover control targets in B2009, composite measure (CM) is defined to examine how close each influenced state is to the desired state of control goal (Fig. 3). CM is defined by *sgn*(*S*_ERK_) × *sgn*(*S*_AKT_) × (1 + *CD*), where *sgn* is sign function, and *CD* is cosine distance between (*S*_ERK_, *S*_AKT_) and (*G*_ERK_, *G*_AKT_) = (+1, +1). The normalization of the influence values is computed by *sgn*(*S*_*ij*_)∙log_2_(1 + |*S*_*ij*_|/*S*_*avg*_), where *S*_*ij*_ is the influence of node *j* on the output *i*, and *S*_*avg*_ is the average of *S*_*ij*_ under the given condition.

In the signal flow analysis (Fig. 3c–f), inhibition of GAB1, IRS, GS, or PDK1 means down-regulation of each of these targets. To implement such inhibition in the signal flow analysis (Fig. 3c–f), we set the basal activity of each control target candidate to −10 without changing any link weight. The hyperparameters, *α* and *β*, were set to 0.5 in the Eq. (1), and link weight normalization is applied (see also Fig. 7b and Table 3).

### Determination of link signs in C2016

In the network of C2016 (Table 1), there are 51 links whose signs can be plus or minus depending on biological contexts. We converted all the undetermined signs of links as plus signs while assuming the incompleteness of curated signaling networks to some extent.

### Implementation of mutation effects

To apply the mutation effects to the networks of B2009, F2013 and C2016 (Table 1), the link weights of some nodes were adjusted. In B2009, the in-links of the mutant protein (RAS or PI3K) were removed to reflect constitutively activating mutations; the weights of out-links of the mutant protein were increased by 10 times. In F2013 and C2016, the weights of in- and out-links of APC, PTEN, and p53 are decreased by 99.9% (to accommodate the attenuation of protein functions); the weights of in- and out-links of Ras are multiplied by 0 and 100, respectively (to accommodate the effect of constitutive activation). In F2013, the link weights of Smad are also adjusted in addition to APC, Ras, PTEN, and p53: the weights of in- and out-links of Smad are multiplied by 0 and 0.01, respectively (for the deletion of Smad4).

### Robustness analysis of SI-plot analysis

In the robustness analysis of SI-plot analysis, the control target candidates were discovered by collecting the top-10% most influential nodes in each SPLO group. The structural perturbation in the robustness analysis was achieved by randomizing the connections between the nodes (i.e., degree-preserving link swapping) or randomizing the signs of links (i.e., activation or inhibition) in the original network^41^. We defined an integer parameter, ‘perturbation’, by which the number of trials to apply the structural perturbation is proportionally increased. The left panels of Fig. 8 show that the number of new links different from the links of the original network (*N*_*DL*_) is increased by the perturbation parameter with some variances. *N*_*DL*_ is mathematically defined as ∑|*A*_*ij*_ − *B*_*ij*_|, where *A* and *B* are the adjacency matrices of the original and perturbed networks, respectively.

The structural perturbations in Fig. 8b,c were defined to always include the links of the critical nodes such as the original control targets or outputs. In the degree-preserving link swapping, we randomly select one link from the links of the critical nodes, and another link from the remaining nodes of networks. Then, we swap the target nodes of the selected links. In the sign flipping, we randomly select one link from the links of the critical nodes and then change its sign (i.e., positive to negative or vice versa). In Fig. 8b, the 8 original control targets, B_Arrestin, beta-catenin, Erk, Mek, p115RhoGEF, PP2A, smad2_4 and Snail, were defined as critical nodes. In Fig. 8c, the 3 output nodes, Apoptosis, Proliferation and Metastasis, were defined as critical nodes.

To measure the robustness of SI-plot analysis with a statistical significance, we defined an enrichment score based on hypergeometric test. In Python, the enrichment score is calculated using NumPy^60^ and SciPy^61^ as follows:

import numpy as np

import scipy as sp

M = 200 # Total number of nodes in the network

n = 8 # Number of the original control targets

N = 56 # Number of the target candidates suggested by SI-plot analysis

k = 6 # Number of the original control targets found by SI-plot analysis

p_value = sp.stats.hypergeom.sf(k-1, M, n, N) # 6.699E-03

enrichment_score = −np.log10(p_value) # 2.174

The enrichment score can quantitatively indicate the robustness of SI-plot analysis with a statistical significance since it is mathematically defined as the negative logarithm of p-value that is calculated by the hypergeometric test.

We chose C2016 for robustness analysis among the signaling networks that have the original control targets (Table 1, B2009, S2015, Z2015, F2013 and C2016) since the size of the network (i.e., the number of nodes and the number of links) is large enough to tolerate the minimal structural perturbation and to have a reasonable statistical significance in the enrichment analysis. For the other networks, we found that an incorrect single link can even cause opposite output values in a small network such as B2009 network in our previous study^41^. We also found that it is difficult to obtain a statistical significance from small networks with respect to the original control targets such as S2015, Z2015 and F2013, since the sampling size of the control target candidates in SI-plot analysis (i.e., a set of the top-*k* or top-*n*% nodes in each SPLO group) is relatively large against the population size of nodes in these small networks.

### Generation of a subnetwork in T2016

We obtained a subnetwork by identifying the giant component of the directed and signed links from the original database of T2016 (Table 1). First, we filtered out the non-directed links (i.e., ‘is_directed’ is 0), and obtained a subnetwork of the single signed links (i.e., either ‘is_stimulation’ or ‘is_inhibition’ is 1) from all interactions of OmniPath (http://omnipathdb.org/interactions). Thereafter, we generated the giant component of the links to obtain the largest connected subnetwork. No output was defined in T2016 since the network was used to examine the performance of the algorithm. The visualization of T2016 network was performed using Cytoscape 3.7.0 (Supplementary Fig. S13a)^62^.

### Performance test

The influence estimation algorithm of SFC was optimized using a GPU device. The CPU and GPU versions of the algorithm were implemented using NumPy^60^ and CuPy^63^, respectively. *α*, *β*, and tolerance parameter (i.e., *tol*) in the iterative method of the algorithm were set as 0.9, 0.1, and 1E-6, respectively. We analyzed the performance of each implementation using a desktop machine where Intel® Core™ i7-4770 CPU (3.40 GHz, 4 cores), Samsung® 32GB DDR3 RAM (4 × 8GB PC3-12800), ZOTAC GeForce® GTX 1080 Ti AMP Extreme (1.645-1.759 GHz, 3584 CUDA cores, 11 GB GDDR5X VRAM), and Microsoft Windows 10 Pro 64-bit operating system are installed.

### Implementation and visualization

We implemented Python package for efficient computation of the influence and SPLO (https://github.com/dwgoon/sfa). To compute SPLO of each node, we utilized the functionality of NetworkX Python package (e.g., networkx.shortest_paths.shortest_path_length)^64^. SI-plot was implemented based on Matplotlib Python package^65^.

## Supplementary information

Supplementary Information

## Data Availability

We provide a GitHub repository for utilization of the algorithm and datasets in a convenient way (https://github.com/dwgoon/sfa). All the networks analyzed in this study are also included in the GitHub repository (SIF format file and NetworkX Python object).
