# Supplementary material for: Signal flow control of complex signaling networks
Source: Sci Rep. 2019 Oct 3;9:14289. doi: 10.1038/s41598-019-50790-0 (PMC6776529; doi:10.1038/s41598-019-50790-0)
Supplement: Supplementary file 1 — Supplementary Information [file 41598_2019_50790_MOESM1_ESM.pdf]

# Supplementary Information

# Signal flow control of complex signaling networks

Daewon Lee and Kwang-Hyun Cho*

Department of Bio and Brain Engineering,
Korea Advanced Institute of Science and Technology (KAIST),
291 Daehak-ro, Yuseong-gu, Daejeon, 34141, Republic of Korea

*Corresponding author. E-mail: ckh@kaist.ac.kr, Phone: +82-42-350-4325, Fax: +82-42-350-4310, Web: http://sbie.kaist.ac.kr/

# Supplementary Figures

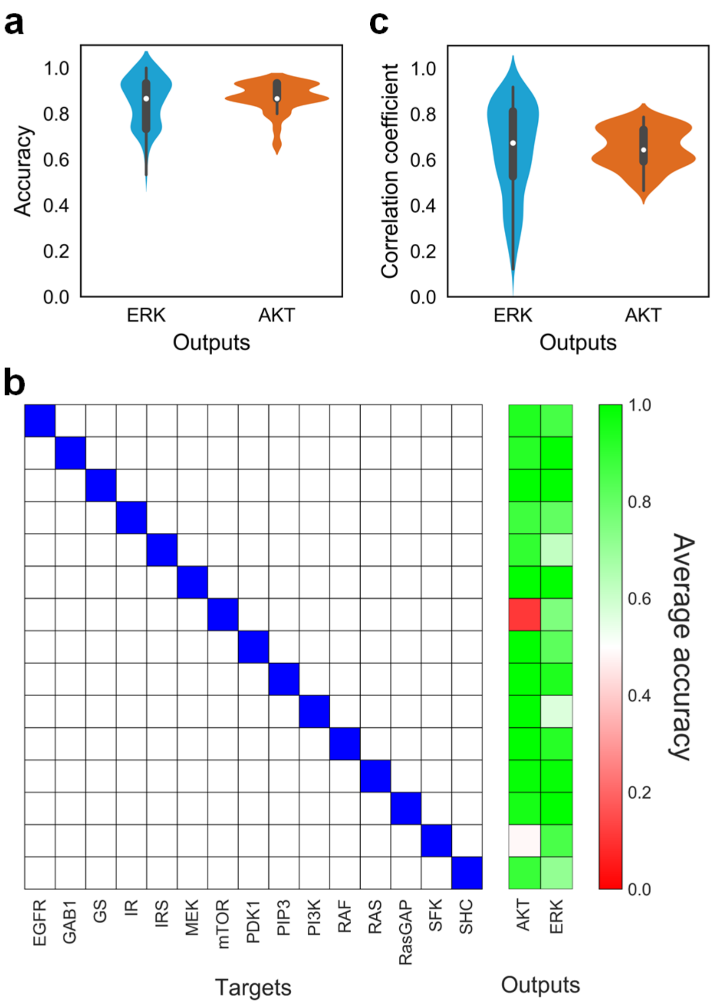


**Supplementary Figure S1. Validation of the estimated influence as a predictive measure. (a)** Distribution of acccuracy for predicting the directions of the activity level changes of ERK and AKT using the signs of influence. **(b)** Average accuracy of the prediction in (a) for each perturbation condition. The accuracy of the mTOR perturbation is the lowest. **(c)** Distribution of Pearson correlation coefficient between the influence and the log-activity change of the original ODE model.

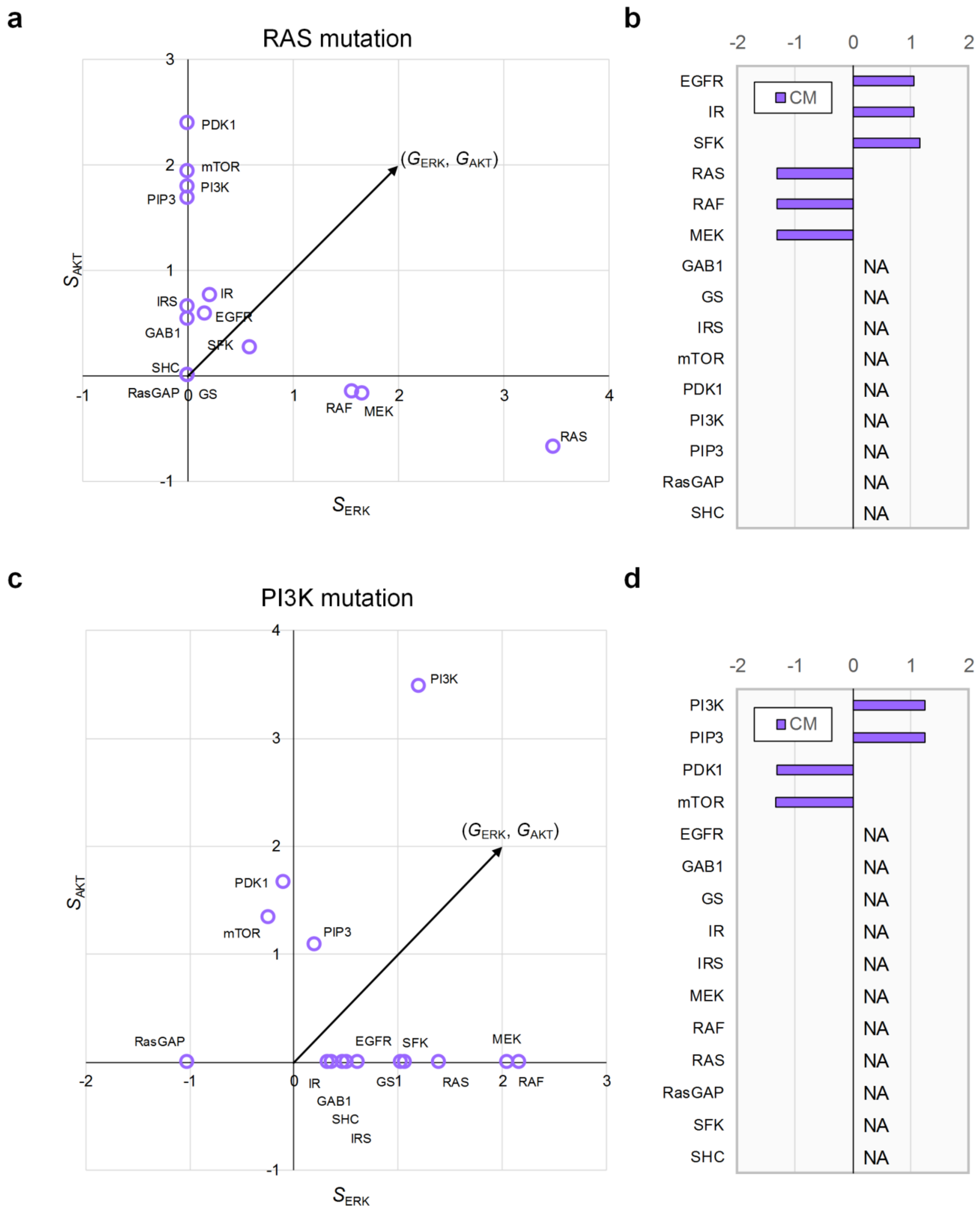


**Supplementary Figure S2. 2D influence space and composite measure in consideration of mutations.** The 2D influence space of ERK and AKT and the composite measure in B2009 in the condition of **(a, b)** RAS mutation, and **(c, d)** PI3K mutation. ($G_{ERK}$, $G_{AKT}$) represents the goal state, and CM is the composite measure (see **Figure 2**). NA in (b) and (d) denotes 'not available'.

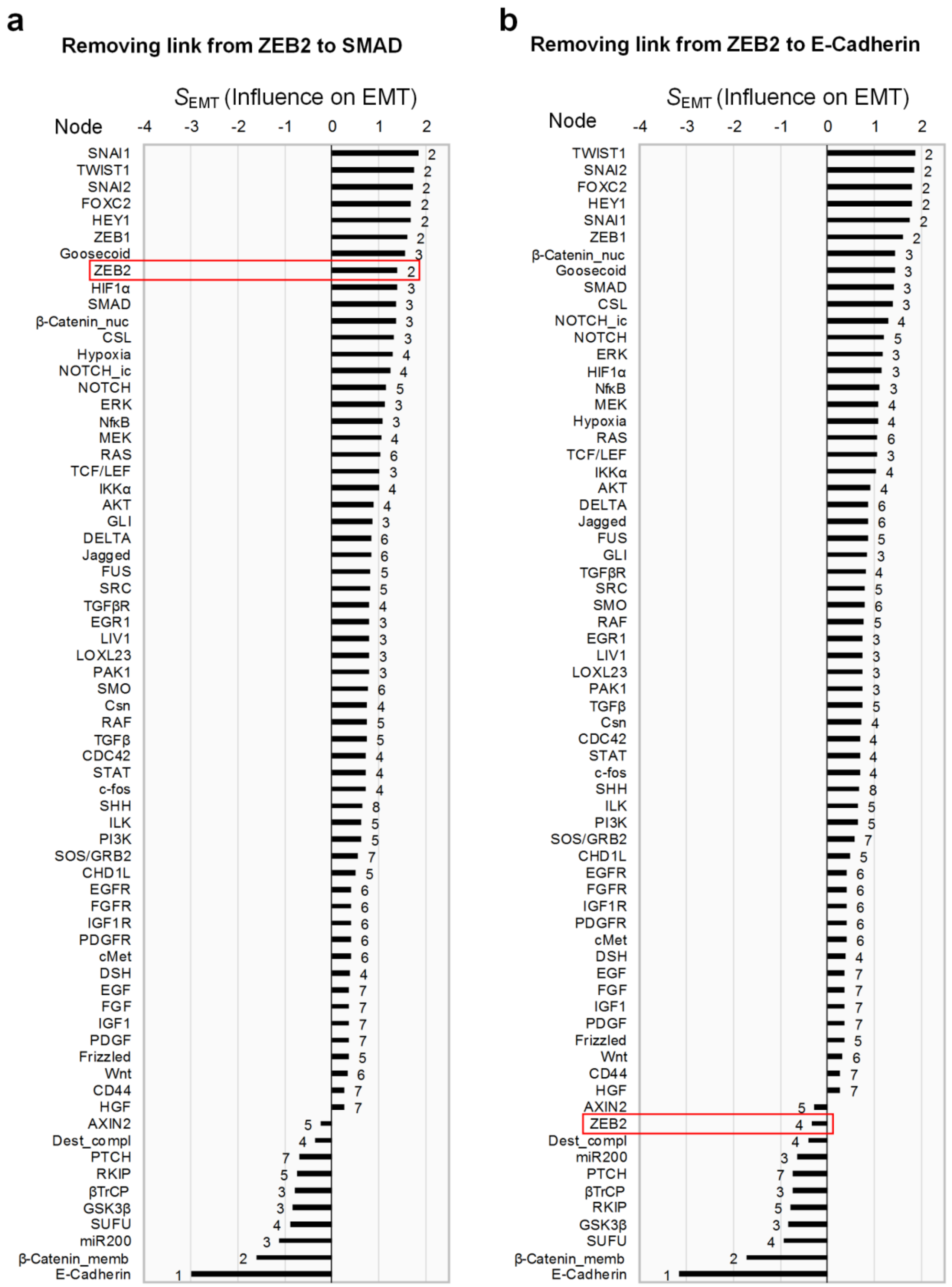


**Supplementary Figure S3. Influences of ZEB2 changed by removing the links in S2015.** Influences on EMT in the condition of removing the link **(a)** from ZEB2 to SMAD, and **(b)** from ZEB2 to E-Cadherin.

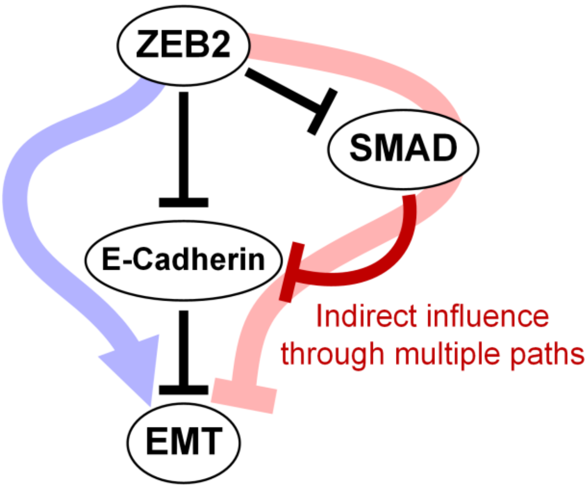


**Supplementary Figure S4. Incoherent feedforward loop (I-FFL) composed of ZEB2, SMAD, and E-Cadherin.** In the I-FFL, ZEB2 directly inhibits E-cadherin, and positively regulates E-Cadherin through SMAD, which creates incoherent influences on the output, EMT.

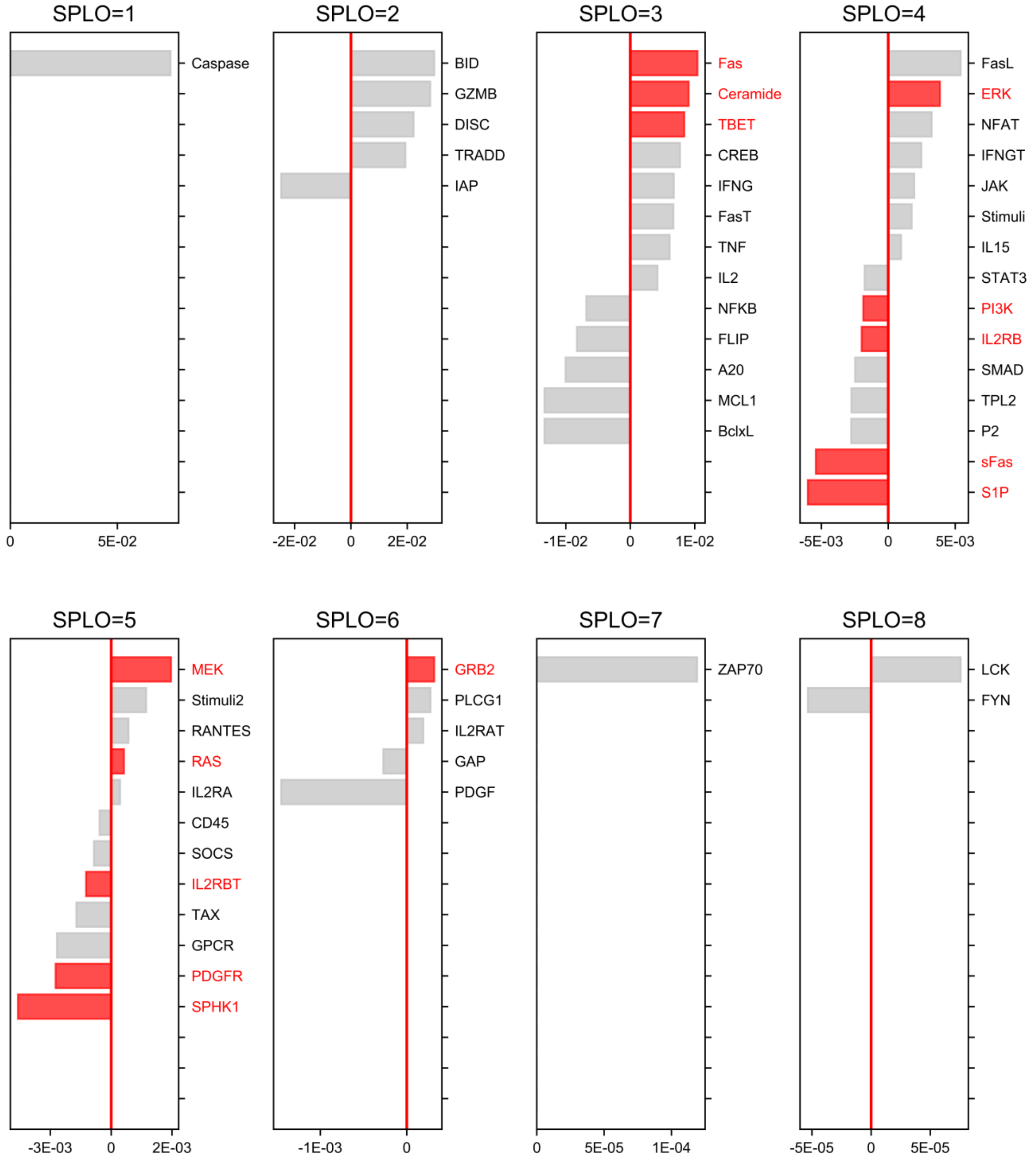


**Supplementary Figure S5. SI-plot of apoptosis in Z2015.** Red denotes the original control targets discovered by stable motif control in Z2015.

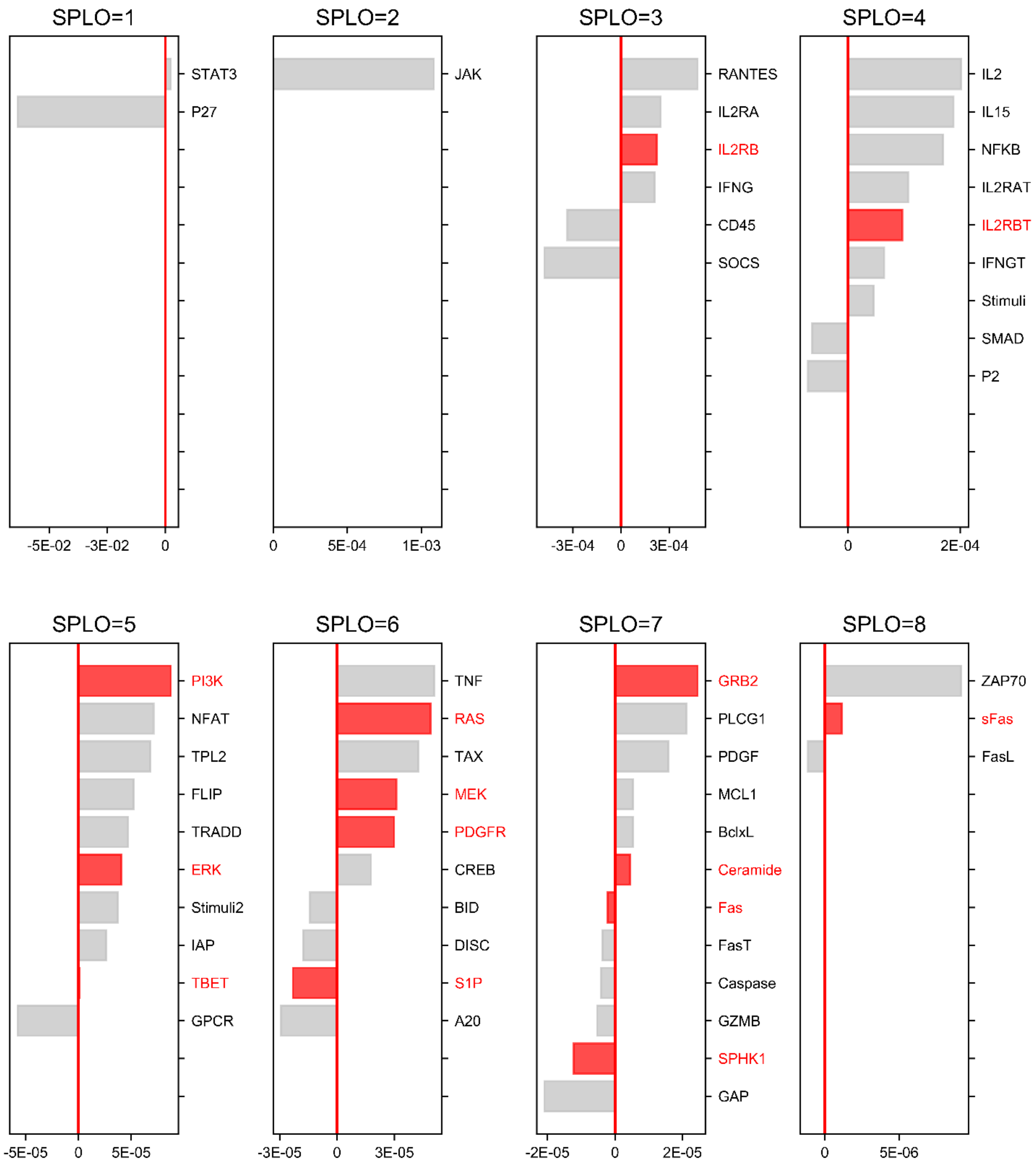


**Supplementary Figure S6. SI-plot of proliferation in Z2015.** Red denotes the original control targets discovered by stable motif control in Z2015.

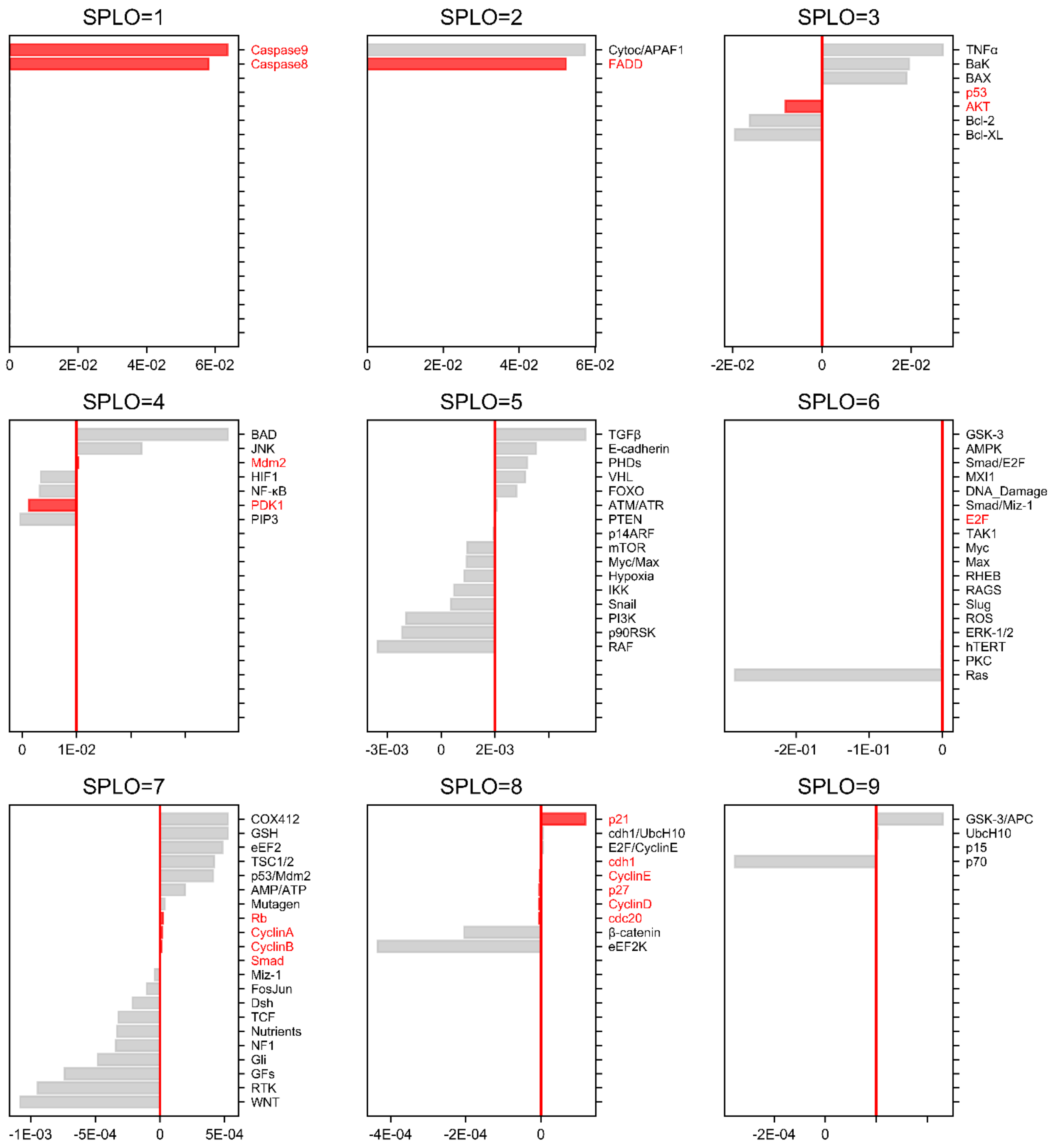


**Supplementary Figure S7. SI-plot of apoptosis in F2013.** Red denotes the original control targets discovered by full search in F2013.

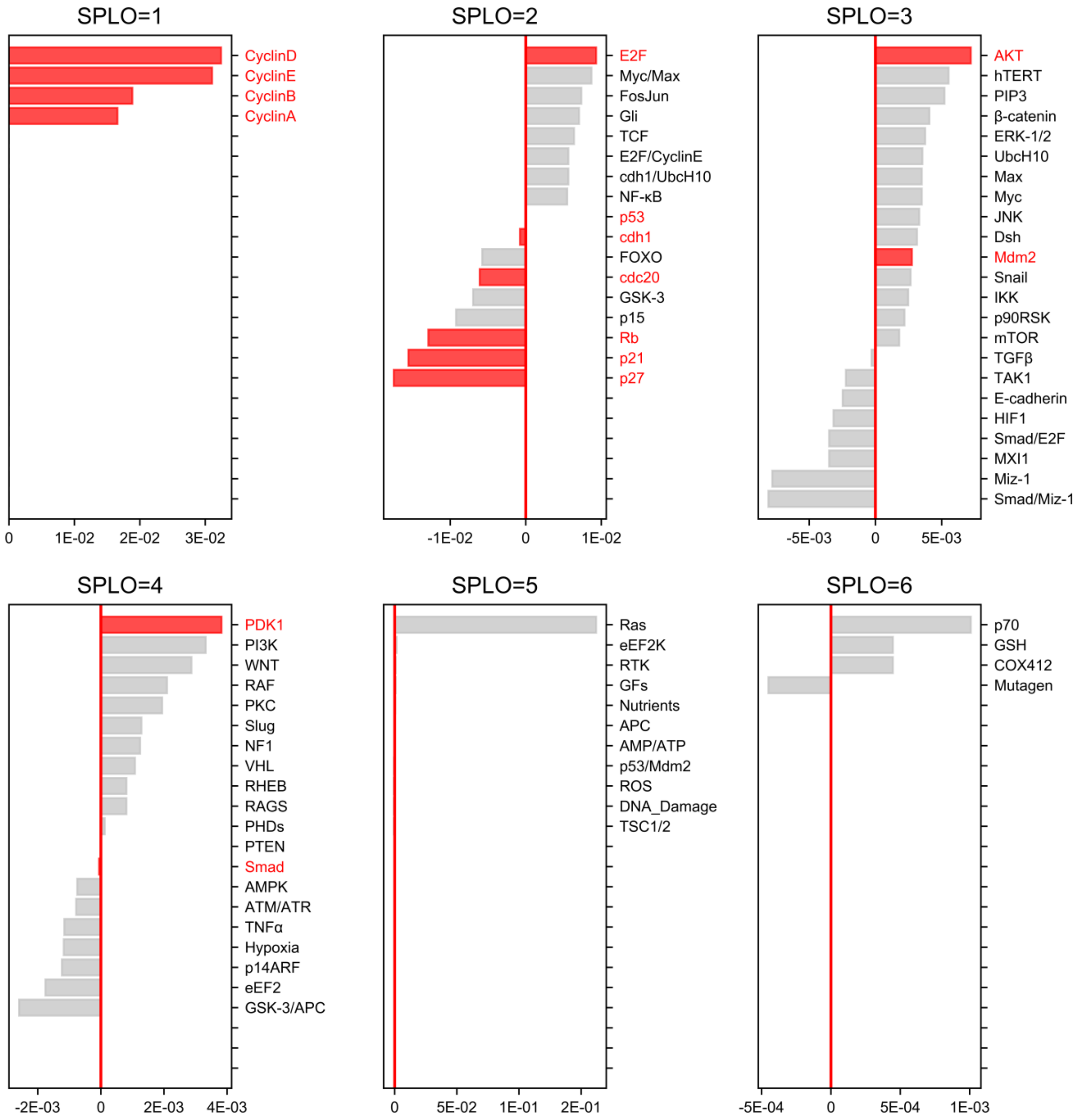


**Supplementary Figure S8. SI-plot of proliferation in F2013.** Red denotes the original control targets discovered by full search in F2013.

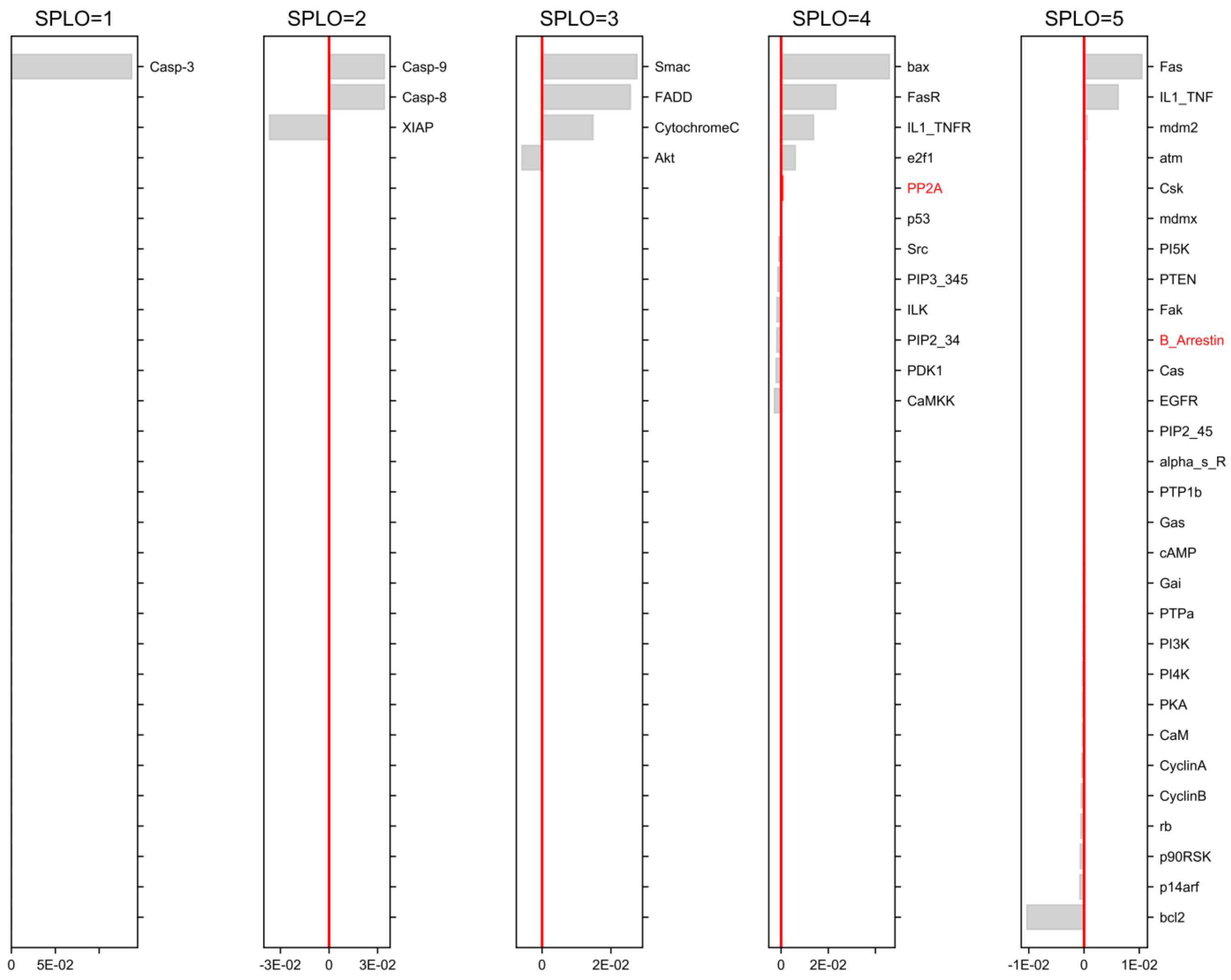


**Supplementary Figure S9. SI-plot of apoptosis in C2016.** Red denotes the original control targets discovered by genetic algorithm optimization in C2016.

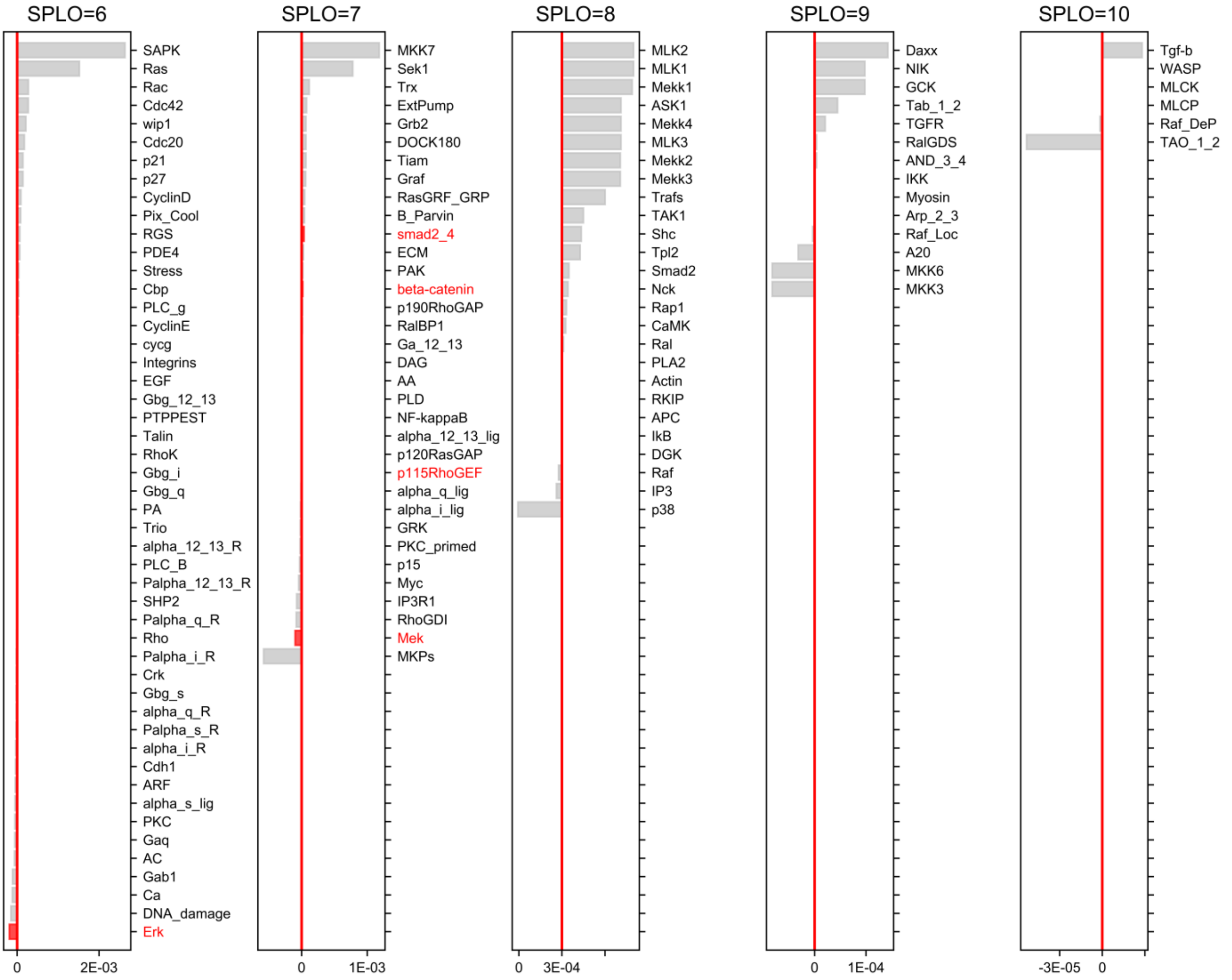


**Supplementary Figure S9. SI-plot of apoptosis in C2016 (continued).** Red denotes the original control targets discovered by genetic algorithm optimization in C2016.

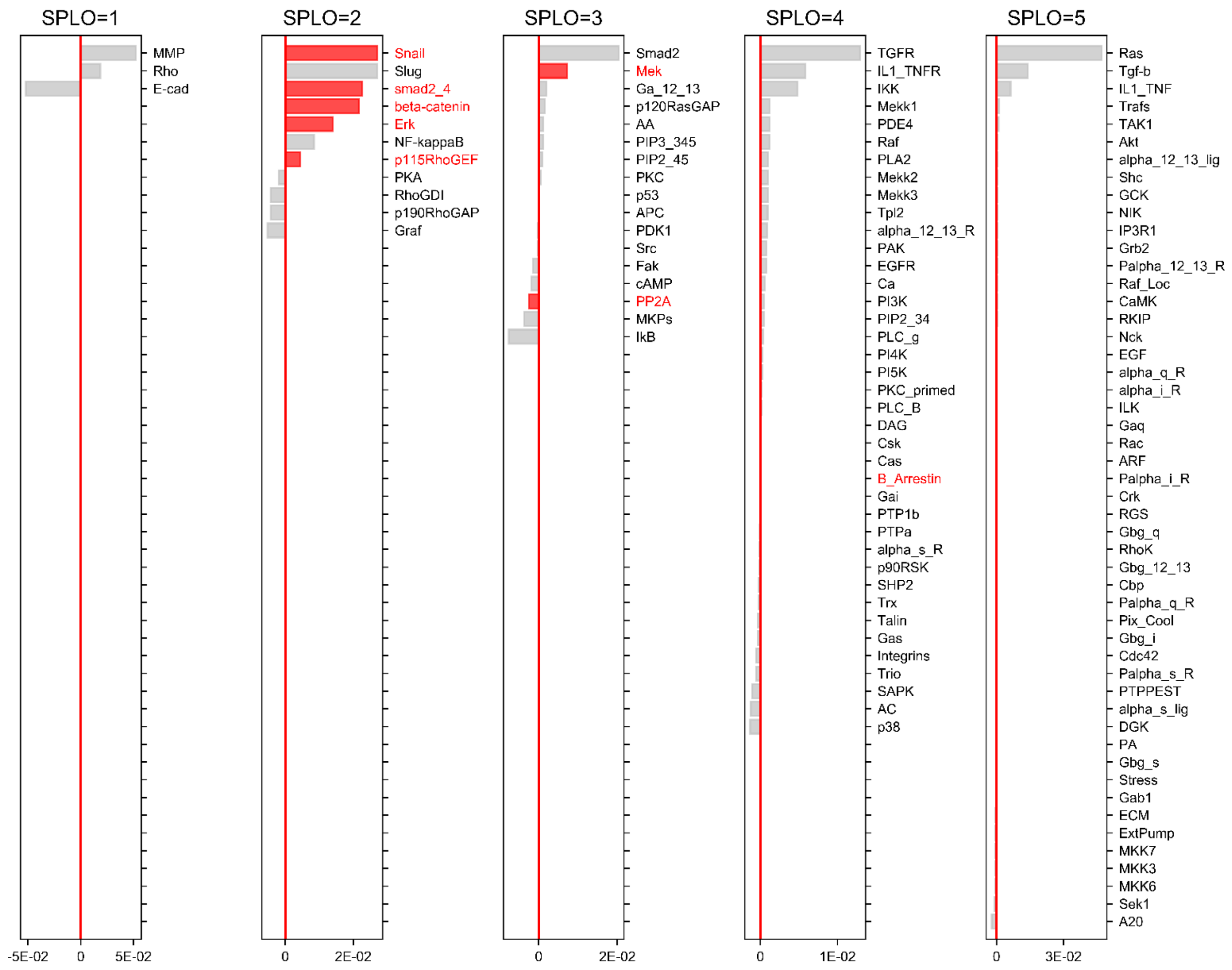


**Supplementary Figure S10. SI-plot of metastasis in C2016.** Red denotes the original control targets discovered by genetic algorithm optimization in C2016.

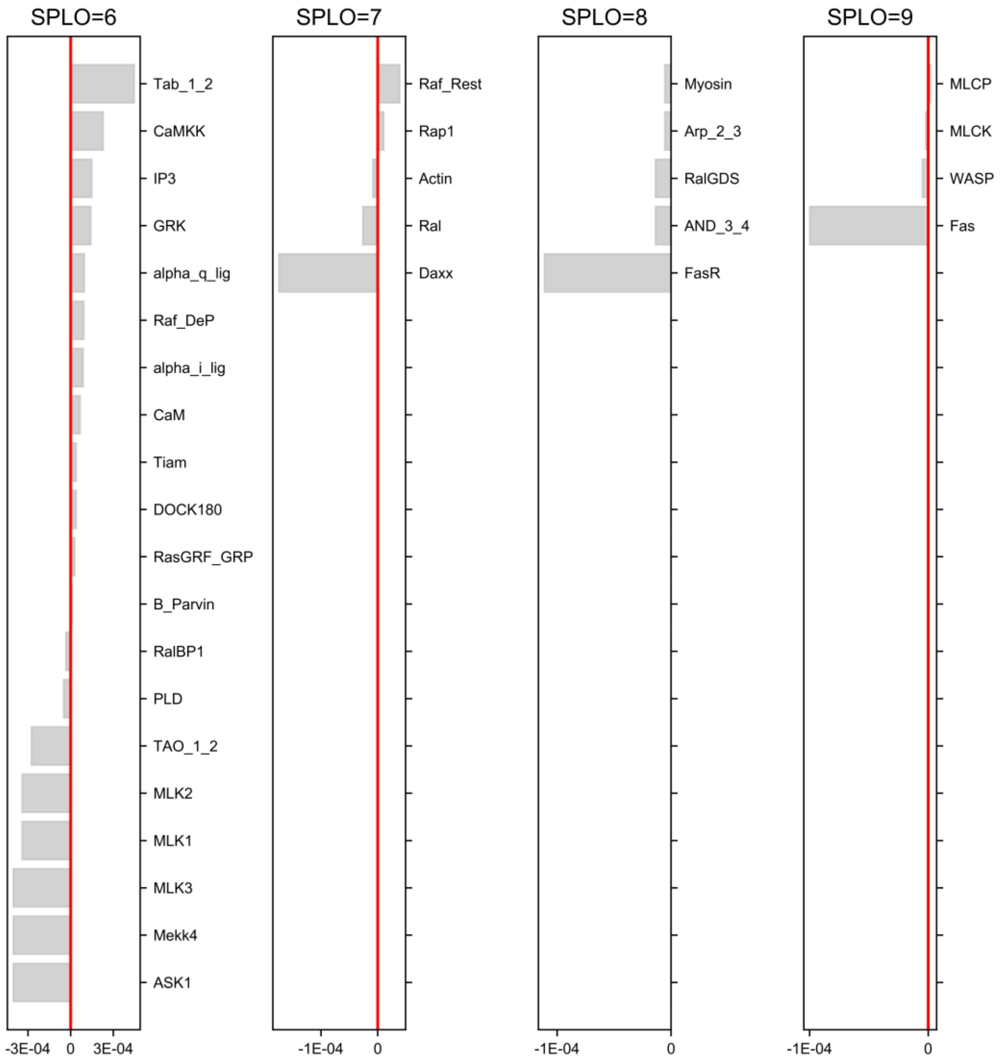


**Supplementary Figure S10. SI-plot of metastasis in C2016 (continued).** Red denotes the original control targets discovered by genetic algorithm optimization in C2016.

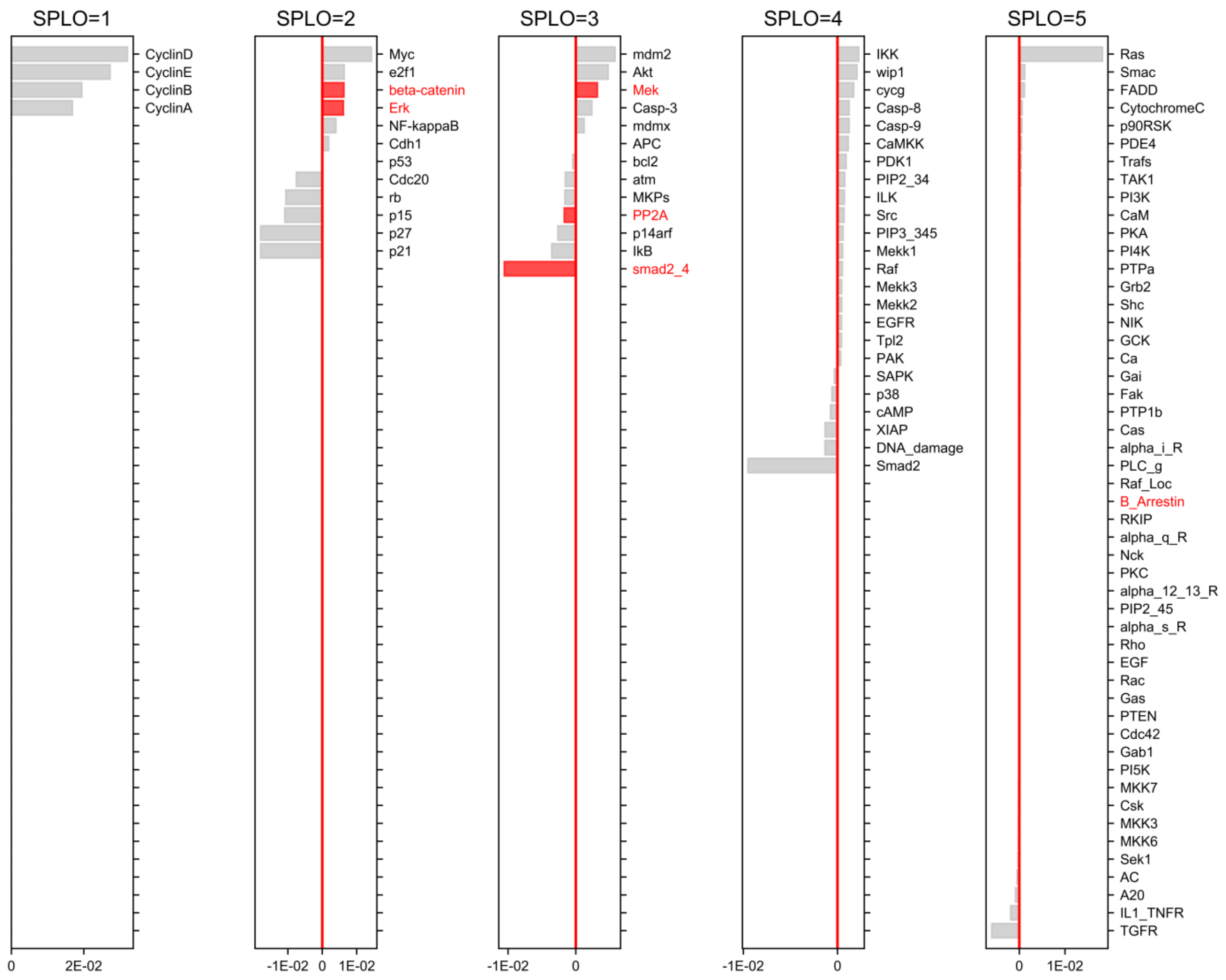


**Supplementary Figure S11. SI-plot of proliferation in C2016.** Red denotes the original control targets discovered by genetic algorithm optimization in C2016.

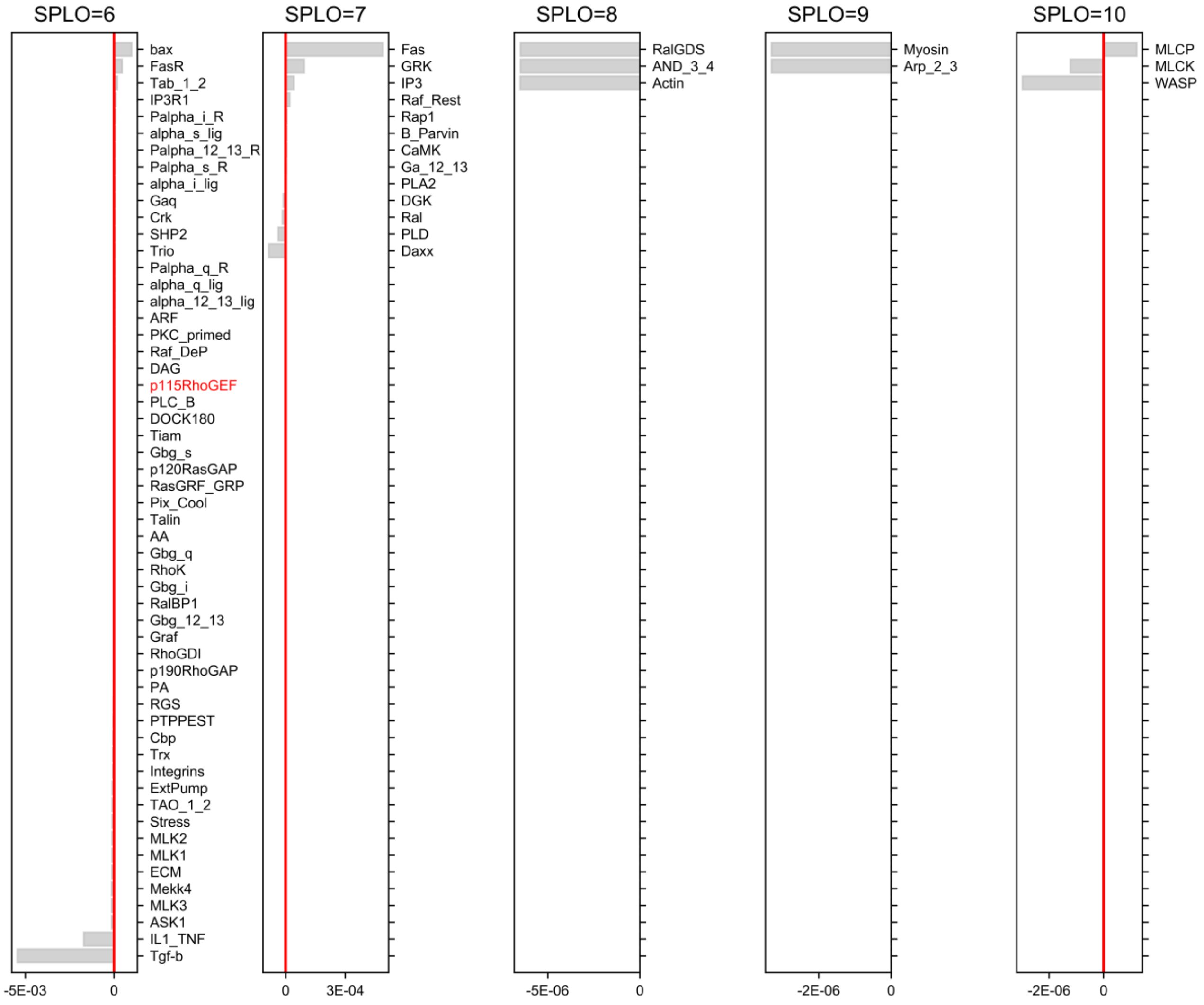


**Supplementary Figure S11. SI-plot of proliferation in C2016 (continued).** Red denotes the original control targets discovered by genetic algorithm optimization in C2016.

a

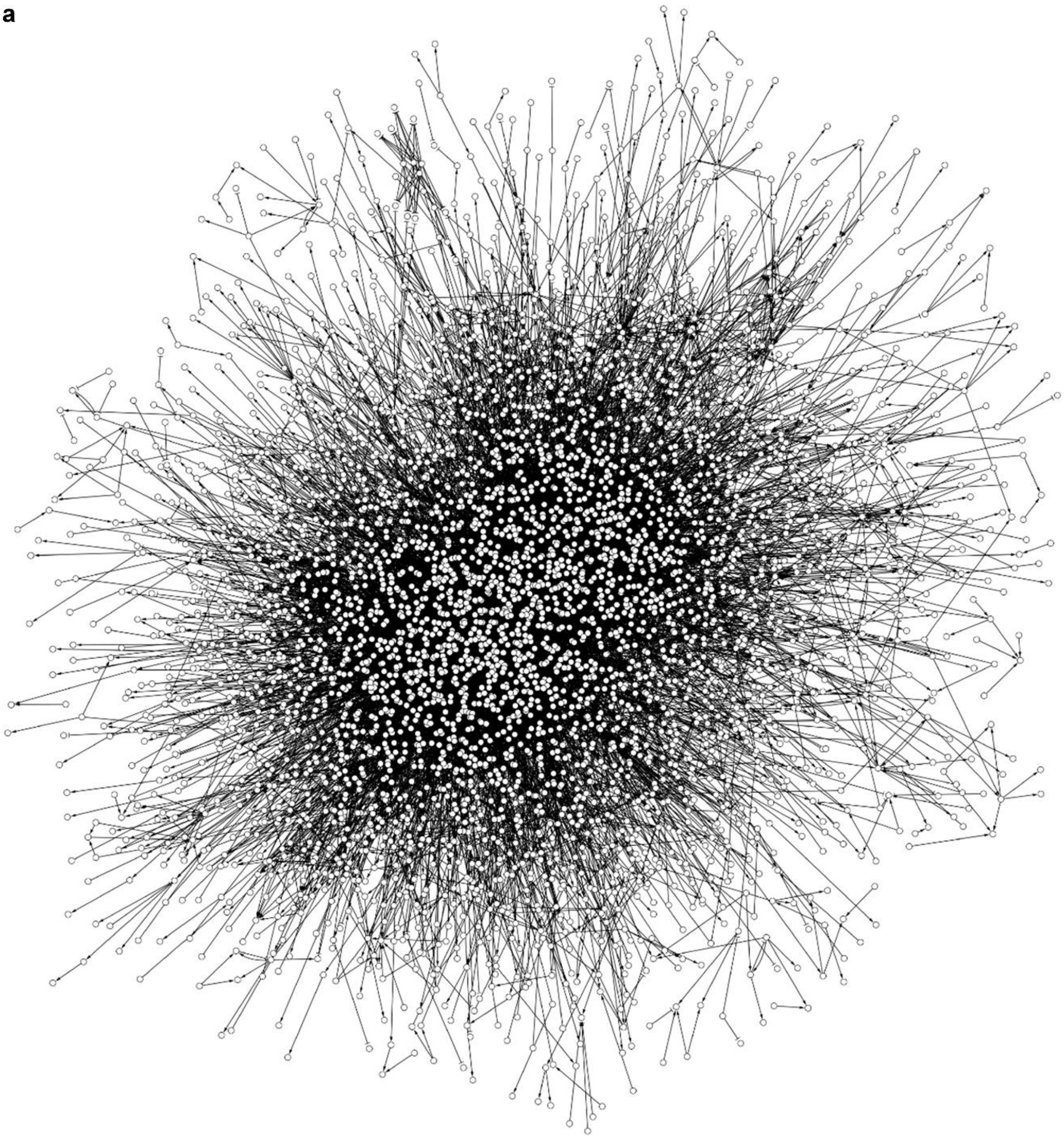

b

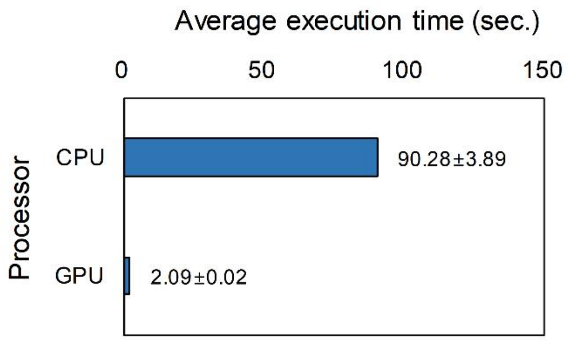


**Supplementary Figure S12. Performance optimization for T2016 network. (a)** Visualization of T2016 network. **(b)** Average duration of 10 repeated executions for the influence estimation of T2016 network. The numbers in the plot denote the average execution time in seconds with standard deviation. See **Methods** in the main manuscript for details.

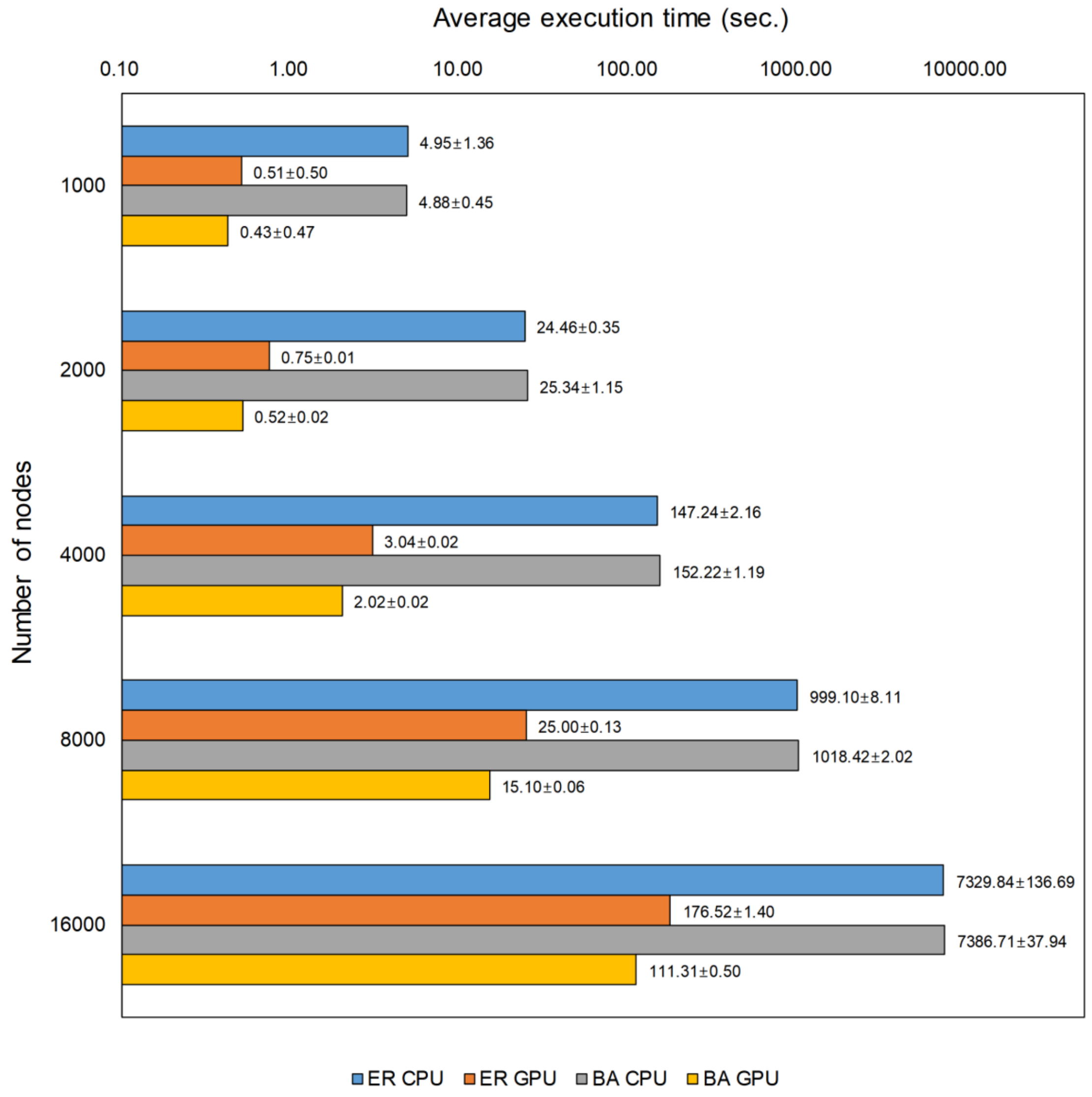


**Supplementary Figure S13. Performance optimization for random networks.** Average execution time was obtained by performing 10 repeated executions of the influence estimation for Erdős–Rényi (ER) and Barabási–Albert (BA) random networks. The probability of edge creation was set as 0.1 in ER networks. The number of edges in BA networks was equal to the number of nodes–1. The numbers in the plot denote the average execution time with standard deviation. See **Methods** in the main manuscript for details.
